# Precise HER2 Protein Degradation via Peptide‐Conjugated Photodynamic Therapy for Enhanced Breast Cancer Immunotherapy

**DOI:** 10.1002/advs.202410778

**Published:** 2024-11-18

**Authors:** Changyong Guo, Fei Gao, Guoyuan Wu, Jinqiu Li, Chunquan Sheng, Shipeng He, Honggang Hu

**Affiliations:** ^1^ School of Medicine or Institute of Translational Medicine Shanghai Engineering Research Center of Organ Repair Shanghai University 99 Shangda Road Shanghai 200444 P. R. China; ^2^ The Center for Basic Research and Innovation of Medicine and Pharmacy (MOE) School of Pharmacy Second Military Medical University (Naval Medical University) 325 Guohe Road Shanghai 200433 P. R. China

**Keywords:** breast cancer, gelatinase responsive peptides, immune induction, photo‐controlled her2 protein degradation, photodynamic immunotherapy

## Abstract

Breast cancer, the most prevalent malignancy among women, frequently exhibits high HER2 expression, making HER2 a critical therapeutic target. Traditional treatments combining the anti‐HER2 antibody trastuzumab with immunotherapy face limitations due to toxicity and tumor microenvironment immunosuppression. This study introduces an innovative strategy combining HER2‐targeting peptides with the photosensitizer (PSs) pyropheophorbide‐a (Pha) via a gelatinase‐cleavable linker, forming self‐assembling nanoparticles. These nanoparticles actively target breast cancer cells and generate reactive oxygen species (ROS) under near‐infrared light, effectively degrading HER2 proteins. Upon internalization, the linker is cleaved, releasing Pha‐PLG and enhancing intracellular photodynamic therapy (PDT). The Pha‐PLG molecules self‐assemble into nanofibers, prolonging circulation, boosting immune induction, and activating CD8^+^ T cells, thus promoting a robust anti‐tumor immune response. In vivo, studies confirm superior biosafety, tumor targeting, and HER2 degradation, with increased cytotoxic T cell activity and improved antitumor immunity. This integrated strategy offers a promising new avenue for breast cancer treatment.

## Graphical abstract

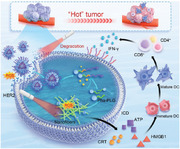

## 1 Introduction

Breast cancer is the most common malignancy among women, with the highest incidence andmortality rates among all types of female cancers.^[^ ^1^, ^2^ ^]^ Human epidermal growth factor receptor 2 (HER2) is a transmembrane glycoprotein that, through dimerization, activates downstream signaling pathways, promoting the proliferation and survival of malignant phenotypes. Consequently, HER2 has become a critical target for the treatment of advanced breast cancer.^[^ ^3^, ^4^ ^]^ Currently, one of the primary treatment methods for breast cancer involves combining the anti‐HER2 antibody trastuzumab with immunotherapy.^[^ ^5^, ^6^ ^]^ However, the efficacy and safety of this combination strategy are significantly limited by the hepatotoxicity and nephrotoxicity associated with traditional drug combinations, as well as the immunosuppressive nature of the tumor microenvironment.^[^ ^7^, ^8^, ^9^, ^10^ ^]^ Therefore, the development of effective HER2‐targeted drug combinations with immunotherapy represents a critical and highly challenging task.

Drugs targeting membrane proteins typically bind to the binding site of the target protein, leading to issues such as acquired mutation resistance or compensatory pathways in the body that confer drug resistance. Targeted protein degradation (TPD) technology, as an emerging drug development strategy, selectively degrades specific proteins, effectively overcoming the limited efficacy and resistance associated with traditional inhibitors, demonstrating significant therapeutic potential.^[^ ^11^, ^12^, ^13^ ^]^ Among these, proteolysis targeting chimeras (PROTACs) have garnered widespread attention for their ability to induce ubiquitination and proteasomal degradation of proteins of interest (POIs).^[^ ^14^, ^15^ ^]^ However, PROTAC relies on the ubiquitin‐proteasome system, which presents limitations in degrading membrane proteins.^[^ ^16^ ^]^ Therefore, developing new strategies for membrane protein degradation not only broadens the scope of TPD technologies but also holds substantial value for drug development.

Current approaches for degrading extracellular and membrane proteins include lysosome‐targeting chimeras (LYTACs),^[^ ^17^ ^]^ cytokine receptor‐targeting chimeras (KineTACs),^[^ ^18^ ^]^ dendritic DNA chimeras (DENTACs),^[^ ^19^ ^]^ and integrin‐facilitated lysosomal degradation (IFLD).^[^ ^20^ ^]^ Although these strategies represent notable advancements, significant challenges persist. Many current methods rely on large molecules, such as bispecific antibodies and aptamers, which are structurally complex, difficult to synthesize, and often lack stability.^[^ ^21^ ^]^ Additionally, the antibodies and oligosaccharide ligands employed in LYTACs can trigger immune responses, and there is uncertainty around the drug‐to‐antibody ratios and conjugation sites for antibody‐drug conjugates.^[^ ^22^ ^]^ Moreover, the broad expression of CI‐M6PR across various cell types and the abundance of liver‐specific ASGPR in normal hepatocytes pose risks; excessive activation of these lysosomal targeting receptors may interfere with physiological functions, potentially causing toxicity and acquired drug resistance.^[^ ^23^ ^]^ This underscores the need for innovative, tumor‐specific membrane protein degradation technologies.

PDT provides a novel approach for HER2 membrane protein degradation by generating ROS from photosensitizers under infrared laser irradiation,^[^ ^24^ ^]^ which can selectively degrade nearby proteins such as PD‐L1^[^ ^25^ ^]^ and BRD4.^[^ ^26^ ^]^ Additionally, PDT can not only directly and specifically destroy tumor cells and tumor neovasculature but also induce immunogenic cell death (ICD) and activate effector T cells, thereby enhancing the immune system's ability to recognize and attack tumors.^[^ ^27^, ^28^ ^]^ However, the limitations of PSs drugs in terms of solubility and targeting significantly restrict their application in the photodegradation of HER2 proteins.^[^ ^29^, ^30^ ^]^

Peptides are increasingly attracting attention in cancer therapy due to their excellent targeting capabilities and solubility.^[^ ^31^, ^32^ ^]^ Conjugating PDT with HER2‐targeting peptides can effectively enhance the solubility and targeting ability of PSs. However, traditional linear peptides suffer from poor stability and are easily degraded by proteases, making it a significant scientific challenge to improve peptide stability.^[^ ^33^ ^]^ Peptide self‐assembly involves the formation of various nanostructures such as nanofibers, nanosheets, and micelles through non‐covalent interactions. Designing self‐assembling peptides or utilizing self‐assembled peptide materials as drug delivery carriers can address issues such as short drug half‐life, poor water solubility, and low physiological barrier penetration.^[^ ^34^, ^35^ ^]^ Furthermore, the phase transition of self‐assembling peptides offers enhanced specificity and targeting toward their protein targets.^[^ ^36^ ^]^ Designing phase‐transitioning peptides specific to HER2 can facilitate better binding to HER2 proteins, inhibit HER2 dimerization, and block the expression of genes related to cell proliferation and survival. This provides an ideal vehicle for the precise photodegradation of HER2 proteins.

This study innovatively combines HER2‐targeting peptides with Pha via a gelatinase‐cleavable linker, forming peptide‐PS conjugate (PPC) that further self‐assemble into nanoparticles. These nanoparticles achieve active targeting of breast cancer cells. Under near‐infrared (NIR) light irradiation, the nanoparticles generate ROS, effectively degrading HER2 proteins on the breast cancer cell membrane. The nanoparticles are then internalized into tumor cells, where the gelatinase cleaves the linker, releasing a PS molecule with three amino acids (Pha‐PLG) and enhancing the PDT effect intracellularly. Additionally, the Pha‐PLG self‐assembles into nanofibers within the cells, prolonging circulation time, enhancing immune induction, and activating a robust immune response by CD8^+^ T cells, thus initiating a powerful tumor‐immune cycle.^[^ ^37^ ^]^ This strategy, which combines HER2 protein degradation with PDT and ICD effects, offers a novel approach to breast cancer treatment.

## 2 Results and Discussion

### 2.1 Design Strategy

**Figure 1:**
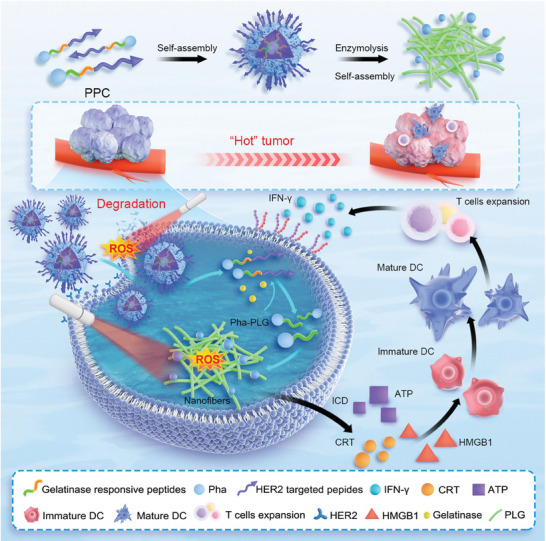
The self‐assembling nanomaterial PPC facilitates photo‐controlled degradation of the HER2 protein in conjunction with PDT, thereby reshaping the tumor microenvironment and augmenting the efficacy of tumor immunotherapy.

In this investigation, a novel compound PPC was synthesized using the solid‐phase peptide synthesis (SPPS), which subsequently self‐assembled into nanospheres (**Figure** **1**). This material is based on Pha and incorporates a gelatinase‐responsive peptide (PLGVRG)^[^ ^38^ ^]^ and an HER2‐targeting peptide (RTYGKRPKIR)^[^ ^39^ ^]^ (PLGVRG‐RTYGKRPKIR is abbreviated as PR) to enable precise targeting and photo‐controlled degradation of HER2, while simultaneously activating an immune response. The unique hydrophobic properties of Pha combined with the hydrophilic nature of the PR facilitate the formation of a hydrophobic core enveloped by a hydrophilic shell, leading to the assembly of classical nanospheres.

Upon cellular interaction, the PR specifically binds to HER2 receptors and collaborates with Pha. Targeted laser irradiation activates the Pha, which reacts with tissue oxygen to generate ROS, driving the photo‐controlled degradation of HER2. Once internalized, the PPC nanomaterials undergo gelatinase‐mediated hydrolysis, resulting in the disassembly of the nanospheres into nanofibers approximately1 µm in length. Due to their larger diameter, these nanofibers are less readily expelled by cells. Empirical evidence indicates that these nanofibers circulate in the body for significantly longer periods compared to their nanosphere counterparts, thereby extending their therapeutic efficacy.

This process triggers ICD response, evidenced by the release of key ICD markers such as adenosine triphosphate (ATP), high mobility group protein B‐1(HMGB1), and calreticulin (CRT). Further in vivo evaluations demonstrate that this self‐assembled material exhibits excellent biosafety, tumor‐targeting efficacy, and substantial HER2 degradation capability. The combination of PPC with PDT strongly induces ICD, leading to the release of ICD signals that facilitate antigen recognition by dendritic cells (DCs). This, in turn, stimulates the secretion of immune‐activating cytokines TNF‐*α* and IL‐6, which are crucial for the maturation of DCs, antigen presentation, and T‐cell activation. Consequently, this altered immune environment leads to a reduction in helper T cells (Floxp3^+^/CD4^+^) and an increase in cytotoxic T cells (CD8^+^), which subsequently secretes IFN‐γ to enhance immune cell activity and drive an antitumor response.

### 2.2 In Vitro Characterization Experiments

**Figure 2:**
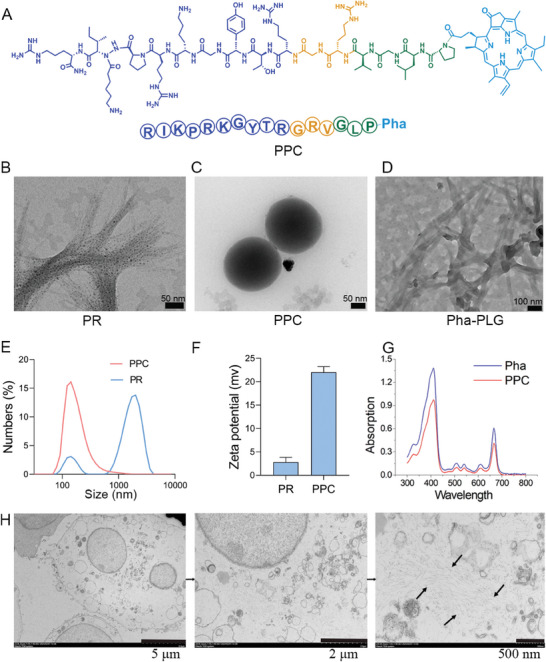
In vitro characterization. A) Chemical structural formula of PPC nanomaterials. B) TEM results of PR. C) TEM results of PPC. D) TEM images of PPC after collagenase digestion. E) DLS measurements of PR and PPC particle sizes. F) DLS measurements of PR and PPC zeta potentials. G) UV–vis absorption spectra of Pha and PPC. H) Bio‐TEM observation of PPC morphology after hydrolysis inside TUBO cells.

To enhance the aqueous solubility, prolong circulatory persistence, and promote targeted delivery of nanomaterials, peptides were synthesized utilizing SPPS (Figure S1, Supporting Information). The chemical structure of the PPC nanomaterials is shown in **Figure** **2**A. In vitro characterization of PR using high‐resolution transmission electron microscopy (TEM) imagery revealed its  morphology as nanofibers, with a length of approximately1 µm (Figure 2B). Subsequently, the self‐assembling nanomaterial PPC was synthesized, displaying a morphology of classical spherical particles with a diameter of appoximately 200 nm (Figure 2C). To replicate the enzymatic milieu in vivo, gelatinase was introduced, eliciting the transformation of PPC into an alternate variant of nanofibers with approximarely1 µm in diameter (Figure 2D), underscoring the extended circulation benefit of PPC. The dimensional attributes and zeta potential of the nanomaterials were appraised by dynamic light scattering (DLS), and the particle size results were corroborated by TEM observations. The PR nanomaterials exhibited dual absorption peaks—a predominant and a minor peak—perfectly mirroring their morphological characteristics (Figure 2E). Both PR and PPC represent alternative forms within anhydrous systems (Figure S2, Supporting Information). The PR displayed a zeta potential of +2.8 mV, whereas the PPC demonstrated a +22.0 mV (Figure 2F). The morphology of PPC was examined before and after enzymatic hydrolysis using a high‐resolution fiberscope (Figure S3, Supporting Information). The absorption peak changes before and after PPC enzymatic hydrolysis were assessed via HPLC, and the molecular weight of the compound following PPC enzymatic hydrolysis was determined using mass spectrometry (Figures S4 and S5, Supporting Information). A UV–vis spectrophotometer performed comprehensive wavelength scans on Pha and PPC, presenting practically congruent principal absorption peaks for PPC and Pha. The primary peaks at 400 and 660 nm were congruent, verifying the successful encapsulation of PR (Figure 2G). Ultimately, bio‐TEM captured the morphological transformation of PPC after 8 hours of cellular assimilation, revealing the conversion of PPC into nanofibers post‐enzymatic hydrolysis within the cellular environment (Figure 2H), which closely paralleled the in vitro observations of collagenase‐induced hydrolysis. These larger nanofibers encounter obstacles during cellular efflux, suggesting a potential bettefit in extending the circulation time.

### 2.3 In Vitro Cytotoxicity Testing and Photocaged Nanomaterial PPC‐Mediated Degradation of HER2 Protein

**Figure 3:**
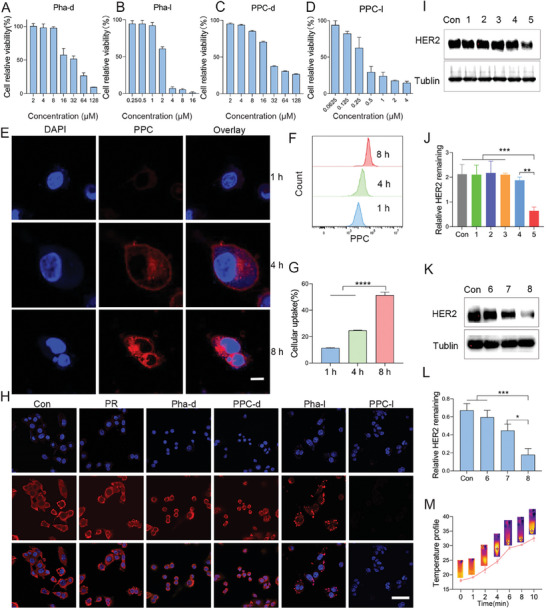
In vitro toxicity, targeting testing, and light‐controlled protein degradation of Pha and PPC. A) CCK‐8 assay for cytotoxicity of different concentrations of Pha‐dark TUBO cells. B)The cytotoxicity of various concentrations of Pha‐light was evaluated using the CCK‐8 assay on TUBO cells with treatments applied using a 660 nm laser 6 min exposure. C) CCK‐8 assay for cytotoxicity of different concentrations of PPC‐dark TUBO cells. D) The cytotoxicity of various concentrations of PPC on TUBO cells was assessed using the CCK‐8 assay, and treatments were applied using a 660 nm laser 6 min exposure. E) Laser confocal microscopy observation of targeting effect of PPC, scale bar: 40 µm. F) Flow cytometry testing of cellular uptake of PPC at different time points. G) Quantitative analysis of cellular targeting of PPC using flow cytometry. Data are presented as mean ± SD (*n* = 3). H) Primary antibodies targeting the HER2 protein were applied, followed by incubation with fluorescent secondary antibodies. Laser confocal microscopy observation of HER2 protein degradation in TUBO cells in different groups, scale bar: 50 µm. I) Western blotting analysis of HER2 protein degradation in different groups of TUBO cells. 1 represents 0.5 µm PR. 2 represents 0.5 µm Pha‐dark. 3 represents 0.5 µm PPC‐dark. 4 represents 0.5 µm Pha‐light. 5 represents 0.5 µm PPC‐light. J) The result of quantitative analysis of HER2 protein degradation in different groups of TUBO cells. Data are presented as mean ± SD (*n* = 3). K) HER2 protein degradation in TUBO cells at different concentrations of PPC. 6 represents 0.13 µm PPC‐light. 7 represents 0.25 µm. 8 represents 0.5 µm PPC‐light. L) The result of quantitative analysis of HER2 protein degradation in different groups of TUBO cells. Data are presented as mean ± SD (*n* = 3). M) Temperature change curve during different laser irradiation measured using an infrared thermometer. Data are presented as mean ± SD (*n* = 3). Statistical significance was calculated using a one‐way analysis of variance. ^*^ *p* < 0.05, ^**^ *p *< 0.01, ^***^ *p *< 0.001.

In vitro cytotoxicity and drug targeting tests were conducted to evaluate the cytotoxic effects of Pha and PPC nanoparticles on TUBO cells. To demonstrate the cytotoxicity of Pha and PPC nanomaterials on tumor cells, a CCK8 assay was utilized to determine the IC_50_ values of Pha and PPC against TUBO cells. Remarkably, our findings revealed that Pha and PPC nanomaterials exerted concentration‐dependent cytotoxic effects solely on the TUBO cells. The IC_50_ values for Pha‐dark were determined as 29.85 and 2.21 µm for Pha‐light, highlighting a 13‐fold distinction in photodark toxicity (**Figure** **3**A,B). In the case of PPC, the IC_50_ values were 30.88 µM for PPC‐dark and 0.68 µm for PPC‐light, indicating a 45‐fold difference in photodark toxicity. (Figure 3C,D). Notably, at 0.5 µm under light conditions, PPC‐light exhibited significant cytotoxicity, highlighting the enhanced cytotoxicity of the drug with the inclusion of targeted peptides.

Subsequently, we investigated the specific localization and cytotoxicity of PPC‐targeting tumor cells. High‐resolution laser confocal microscopy was employed to observe changes in cell localization following nanomaterial treatment at different time points. PPC was initially localized to the cell membrane within the first 4 h and subsequently transitioned to the intracellular region after 4 h, with intracellular content gradually increasing over time (Figure 3E). Additionally, the cellular uptake of PPC over time was quantified using flow cytometry (Figure 3F) and further subjected to quantitative analysis (Figure 3G). To assess the targeting specificity of PPC, we performed flow cytometry experiments to evaluate the binding of HER2‐targeting peptides on HER2‐positive TUBO cells and HER2‐negative 4T1 cells. The results showed that PPC effectively targeted HER2‐positive TUBO cells while demonstrating only weak binding to HER2‐negative 4T1 cells. (Figure S6, Supporting Information). In vitro assessments revealed that PPC self‐assembled nanomaterials exhibited favorable biocompatibility, cytotoxicity profiles, and cellular targeting capabilities.

To further assess the degradative impact of the light‐responsive self‐assembling nanomaterial PPC on HER2 protein, TUBO cells were cultured at a density of 2 × 10^5^ in glass dishes. Following a 4 h drug exposure, the cells underwent laser irradiation, and protein expression on the cell membrane was visualized using laser confocal microscopy. The results indicated that PPC‐light demonstrated notable cellular degradation capability, whereas Pha‐light exhibited modest degradative effects, with Pha‐dark and PPC‐dark showing minimal degradative effects (Figure 3H). Fluorescence data revealed a 3.96‐fold increase in the protein degradation capacity of PPC‐light (Figure S7, Supporting Information). Subsequently, western blotting was employed to confirm the degradative potential of the light‐responsive self‐assembling material on HER2 protein(Figure 3I,J). Protein degradation analysis using varying concentrations of PPC‐light revealed that 0.13 and 0.25 µm PPC‐light had negligible impact on protein degradation, while 0.5 µm PPC‐light exhibited HER2 protein degradation capabilities (Figure 3K,L). The HER2 membrane protein was also extracted and incubated with PPC in vitro. Afterward, the samples were exposed to NIR light irradiation. The results showed substantial degradation of the HER2 protein *in* *vitro*, with the degree of degradation closely matching that observed in cellular settings (Figure S8A,B, Supporting Information). To explore the mechanism of PPC‐mediated HER2 protein degradation, we conducted further experiments. We found that light‐induced HER2 degradation was partially reversed with the addition of ROS scavengers. Conversely, in the absence of these scavengers, PPC substantially reduced HER2 protein levels upon NIR light exposure (Figure S8C,D, Supporting Information). These findings suggest that PDT effectively facilitates the degradation of the HER2 receptor protein through a ROS‐mediated mechanism. The expression of estrogen receptor (ER) protein following treatment with PPC‐light was also assessed. The results indicated that PPC‐light had minimal impact on ER protein expression, even under light exposure. The findings suggest that the effects of PPC‐light are mediated through HER2‐specific degradation rather than general disruption of the membrane (Figure S9, Supporting Information). The comprehensive membrane outcomes of protein degradation across various groups were confirmed through western blot analysis (Figure S10, Supporting Information).

Furthermore, to assess whether the thermal effects induced by photodynamic therapy caused cellular damage, we investigated the photothermal efficiency of the therapy. Using a thermal imaging camera, temperature changes over 10 min were monitored, revealing that photodynamic therapy raised the material's temperature from 18 to 33 °C within 10 min‐comparable to body temperature and posing no harm to the cells (Figure 3M). PPC nanomaterials exhibited excellent capability in inducing the degradation of cell membrane proteins through low‐temperature photocontrol.

### 2.4 Experiments on the Generation of ROS and Cell Apoptosis In Vitro

**Figure 4:**
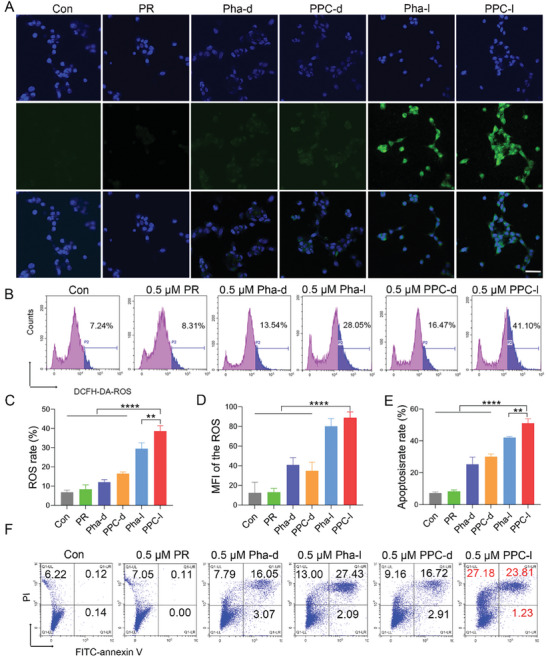
Generation of ROS and cell apoptosis testing. A) Confocal microscopy detection of ROS generation in TUBO cells after different treatments with DCFH‐DA staining, scale bar: 50 µm. B) Flow cytometry analysis of ROS generation. C) Fluorescence intensity analysis of ROS generation observed through confocal microscopy. Data are presented as mean ± SD (*n* = 3). D) Flow cytometric analysis of ROS generation capacity. Data are presented as mean ± SD (*n* = 3). E) The result of quantitative apoptosis rate determination. Data are presented as mean ± SD (*n* = 3). F) Flow cytometry analysis of apoptosis in TUBO cells after staining with Annexin‐FITC and PI, scale bar: 50 µm. Statistical significance was calculated using a one‐way analysis of variance, with ^**^ *p <* 0.01, ^***^ *p <* 0.001, ^****^ *p <* 0.0001.

To investigate the mechanism of cell death, we initially utilized 2,7‐dichlorofluorescein diacetate (DCFH‐DA) to evaluate intracellular ROS levels. At a treatment concentration of 0.5 µm for Pha‐dark and PPC‐dark, intracellular ROS levels were lower, whereas higher ROS levels were observed under Pha‐light and PPC‐light conditions at the same concentration (**Figure** **4**A). Furthermore, quantitative assessment of ROS content via flow cytometry demonstrated that ROS levels in the PPC‐light group were 5.6 times higher than those in the control group and 2.3 times higher than those in the PPC‐dark group (Figure 4B,C). Analysis of confocal fluorescence intensity indicated that ROS content in the PPC‐light group was 7.1 times higher than that in the control group (Figure 4D). Disruption of intracellular redox homeostasis has been linked to severe damage to cellular components such as organelles, lipids, and proteins, ultimately leading to cell death. To further explore the mechanism of cell death, we employed flow cytometry to analyze cell apoptosis using Annexin V‐FITC and propidium iodide (PI) assay kits. The results revealed that the apoptosis rate in the PPC‐light group was 52.22%, surpassing that of the Pha‐light group (42.52%) and the PPC‐dark group (28.79%) (Figure 4E,F). The apoptotic morphological changes in distinct cell groups were observed under a microscope (Figure S11, Supporting Information). In vitro studies demonstrated that PPC nanomaterials were capable of eliciting elevated levels of ROS expression and promoting apoptosis.

### 2.5 In Vitro Immune Induction Experiments

**Figure 5:**
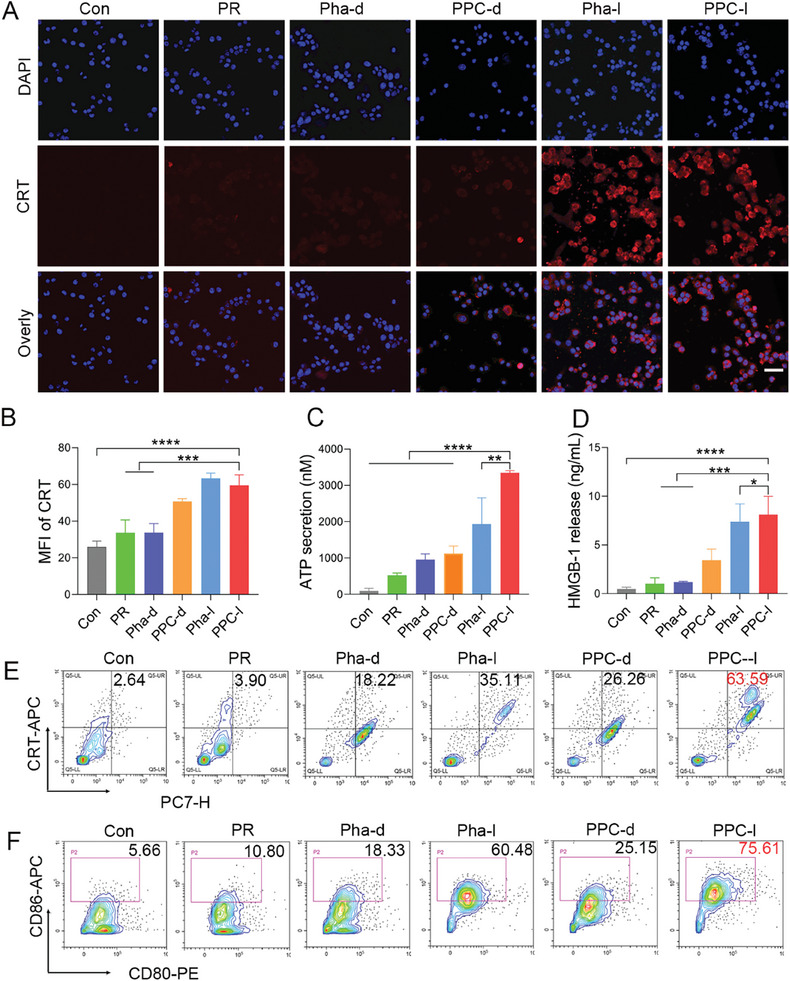
In vitro analysis of ICD induction. A) Confocal microscopy was used to observe the induction of CRT within TUBO cells, scale bar 50 µm. B) Quantitative analysis of the co‐localization of CRT protein by confocal fluorescence microscopy was performed to examine the results. Data are presented as mean ± SD (*n* = 3). C) Assessment of ATP generation within TUBO cells post‐drug treatment. Data are presented as mean ± SD (*n* = 3). D) Evaluation of HMGB1 release capacity within TUBO cells after administration. Data are presented as mean ± SD (*n* = 3). E) Flow cytometry was employed to assess the CRT induction results in TUBO cells post‐treatment, F) The results of in vitro DC induction in each experimental group, scale bar 50 µm. Statistical significance was calculated using a one‐way analysis of variance, with ^**^ *p <* 0.01, ^***^ *p <* 0.001, ^****^ *p <* 0.0001.

Studies have demonstrated that elevated levels of ROS can trigger a robust ICD effect.^[^ ^27^ ^]^ PSs within cells can generate substantial ROS which also possesses ROS release capabilities, and can similarly induce potent ICD effects, thereby activating the immune system to combat tumors. During ICD, damage‐associated molecular markers from immunogenic apoptotic cell fragments, such as ATP, CRT, and HMGB1, are released. PPC‐light can directly eliminate tumor cells, causing the release of intracellular contents and immune‐inducing substances. Using laser confocal microscopy, we initially observed that PPC‐light treatment induced surface levels of calreticulin on cells. The results revealed that the level of CRT protein in the PPC‐light group was 2.3 times higher than those in the control group, serving as a signal to immune cells to “find me” and enhancing the antigen‐presenting cell's role in transmitting signals to immune cells (**Figure** **5**A,B). In fact, compared to the control group, the extracellular ATP secretion in the supernatant of TUBO cells treated with PPC‐light increased by 35‐fold compared to the blank control group and 1.7‐fold compared to Pha‐light (Figure 5C). This released ATP can function as an “eat me” signal, promoting the recruitment of immune cells to the tumor area, thereby triggering ICD. Furthermore, the HMGB1 signal significantly increased after PPC‐light treatment, with a 16‐fold increase in the PPC‐light group compared to the control group (Figure 5D). Flow cytometry analysis confirmed the upregulation of CRT expression. Specifically, the PPC‐light group demonstrated a remarkable 24.1‐fold elevation in CRT levels relative to the control group, whereas the CRT levels in the PPC‐light group were 3.4 times higher than those in the Pha‐dark group (Figure 5E; Figure S12A, Supporting Information). The DC induced by PPC‐light was 4.1‐fold higher than that in the Pha‐dark group and 13.4‐fold higher than that in the control group (Figure 5F; Figure S12B, Supporting Information). We also observed the induction of DC maturation by PPC‐light through microscopy (Figure S13, Supporting Information). The combined induction of ICD in TUBO cells treated with PPC‐light serves as an antigen presentation, promoting the maturation of DCs.

### 2.6 Evaluation of In Vivo Targeting and Inhibition of Tumor Distal Metastasis

**Figure 6:**
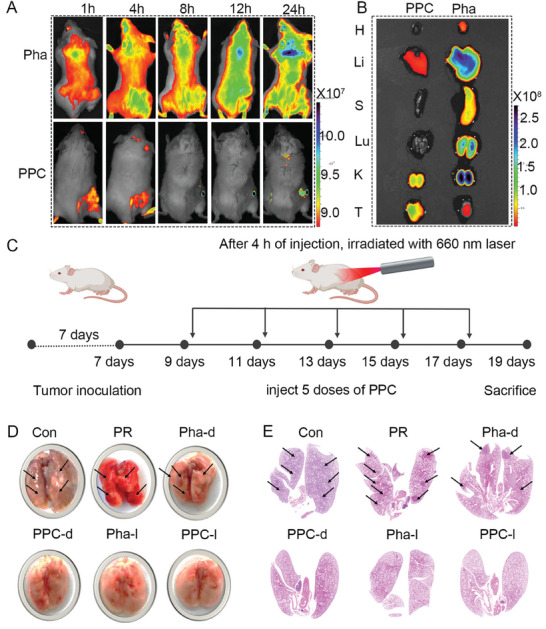
Assessment of in vivo anti‐tumor efficacy. A) in vivo fluorescence imaging of TUBO tumor‐bearing mice at 1, 4, 8, 12, and 24 h post intravenous administration of Pha and PPC. B) *Ex vivo* fluorescence imaging of primary organs and tumors at 24 h. C) Schematic depiction of the establishment of the TUBO tumor model along with detailed experimental protocols. D) Visual representation of lung tumor metastasis. E) *H*&*E* staining images of lung tumor metastasis.

In this study, we initially assessed the tumor‐targeting and distribution characteristics of Pha and PPC through in vivo fluorescence imaging before investigating the anti‐tumor effects of antibodies.Balb/c mice bearing TUBO tumors were intravenously administered Pha and PPC, and the fluorescence signal intensity was compared at various time points. At 8 h post‐injection, PPC exhibited the highest tumor accumulation. Even after 24 h, a strong fluorescence signal was still evident, while free Pha displayed poor tumor targeting and scattered distribution in vivo (**Figure** **6**A). Fluorescence imaging of primary organs and tumors revealed that PPC possessed superior tumor accumulation ability and could undergo metabolic processes in the liver and kidneys (Figure 6B). A schematic diagram illustrated the animal modeling and drug administration protocols (Figure 6C). Infrared imaging was used to verify the temperature changes in mice following PPC administration (Figure S14, Supporting Information). Additionally, the biodistribution data suggested that the optimal therapeutic effects from PDT treatment could be achieved by conducting the treatment 8 h post‐PPC injection.

Subsequently, we investigated whether PPC‐light therapy could effectively inhibit tumor cell metastasis. Treatment with PR and Pha‐dark alone failed to effectively suppress lung metastasis, resulting in prominent lung nodules. Conversely, the Pha‐light, PPC‐dark, and PPC‐light groups demonstrated significant suppression of lung metastasis, with minimal visible lung nodules (Figure 6D). Notably, the PPC‐light treatment group exhibited minimal tumor metastasis based on lung tissue *H*&*E* staining results (Figure 6E). This outcome was primarily attributed to the excellent tumor‐targeting ability of PPC‐light, which, upon exposure to near‐infrared light, produced elevated levels of ROS within the tumor, effectively inducing tumor cell death and triggering a robust tumor immune response.

### 2.7 Evaluation of In Vivo Immune Induction and Anti‐Tumor

**Figure 7:**
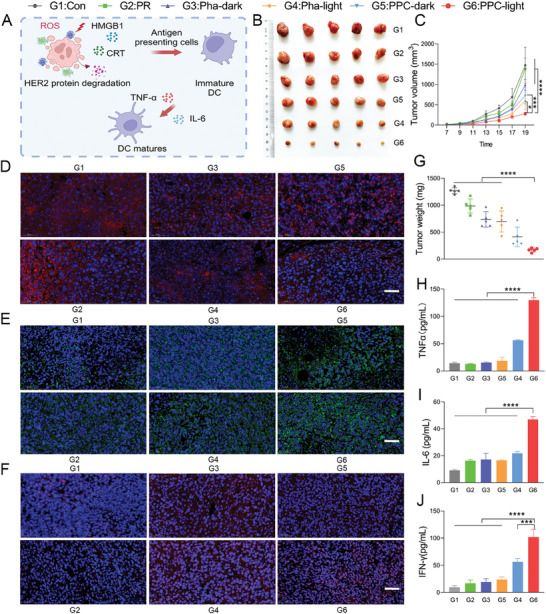
Evaluation of in vivo anti‐tumor efficacy. A) The schematic depicts the maturation of DCs induced by protein degradation and immune factors. B) Visual images of tumors post‐treatment in each group. C) Plot of tumor curves post 5 administrations in each group. Data are presented as mean ± SD (*n* = 5). D) Results of HER2 protein degradation post‐administration, scale bar: 50 µm. E) Induction of CRT in mouse tumors post‐treatment, scale bar: 50 µm. F) Generation of HMGB1 in mouse tumors post‐treatment, scale bar: 50 µm. G) Tumor weight change curve in each group. Data are presented as mean ± SD (*n* = 5). H) ELISA measurement of TNF‐*α* content in mouse serum post‐administration. Data are presented as mean ± SD (*n* = 3). I) Measurement of IL‐6 content in mouse serum following administration using ELISA. Data are presented as mean ± SD (*n* = 3). J) ELISA measurement of IFN−γ content in mouse serum post‐administration. Data are presented as mean ± SD (*n* = 3). Statistical significance was calculated using a one‐way/two‐way analysis of variance, with ^*^ *p <* 0.05, ^**^ *p <* 0.01, ^***^ *p <* 0.001, ^****^ *p <* 0.0001.

Next, we comprehensively evaluated the therapeutic efficacy of PPC in vivo. Given the biocompatibility and tumor‐targeting specificity of PPC, we established a TUBO tumor model in Balb/c mice by subcutaneously inoculating 3 × 10^5^ TUBO cells on the right flank to establish the primary tumor model. The mice were randomly divided into 6 treatment groups: saline(control group), PR, Pha‐dark, Pha‐light, PPC‐dark, and PPC‐light, with intravenous injections administered every 2 days. Tumor size at the primary tumor site was measured with calipers every 2 days, and treatment began when the average tumor size reached 60 mm^3^. A schematic representation illustrated intracellular protein degradation and ICD induction (**Figure** **7**A). Compared to the control group, PR, Pha‐dark, and PPC‐dark groups, the PPC‐light group exhibited a significant inhibition of primary tumor growth (Figure 7B). Statistical analysis of tumor weights across the groups revealed an impressive 86% tumor inhibition rate for the PPC‐light group compared to the control group (Figure 7C). Additionally, the PPC‐light group demonstrated the most successful inhibition of primary tumor growth, as depicted in the tumor volume statistics among the groups.

The study indicated aberrant HER2 protein expression during tumor proliferation. To validate the degradative effect of light‐triggered self‐assembling materials on HER2 protein, tumor immunofluorescence staining was performed after 5 administrations. The results demonstrated that the control, PR, Pha‐dark, and PPC‐dark groups exhibited limited HER2 protein degradation, whereas the PPC‐light group exhibited the strongest degradation effect (Figure 7D). To examine the early induction effect of ICD by self‐assembling nanomaterials, tumor tissues were collected after 3 administrations for immunofluorescent staining of CRT and HMGB1 to assess ICD activation within primary tumors. Immunofluorescent images of CRT and HMGB1 in the primary tumor showed increased CRT expression and strong HMGB1 release induction in the PPC‐light group (Figure 7E,F). Immunohistochemical analysis also confirmed elevated levels of CRT and HMGB1 in the PPC‐light group (Figure S15, Supporting Information). PPC‐light demonstrates robust induction of ICD *in vivo, which* plays a pivotal role in antigen presentation. Throughout the 12‐day observation period, the mice in each treatment group maintained consistent body weights, indicating no biotoxic side effects from the drugs (Figure S16A,Supporting Information). At the same time, the tumor weights were assessed for each group, revealing a significant inhibition of tumor growth by the PPC‐light group (Figure 7G).

Furthermore, to gain deeper insights into the mechanisms enhancing anti‐tumor immunity, peripheral blood serum samples were analyzed post‐5 administrations using enzyme‐linked immunosorbent assay (ELISA) to measure levels of immune‐stimulating cytokines secreted. The results revealed a significant increase in TNFα, IFN‐γ, and IL‐6 levels in the PPC‐light treatment compared to other therapies (Figure 7H–J). These findings suggest that PPC‐light‐mediated self‐assembling nanomaterials effectively facilitate antigen presentation during anti‐tumor immune therapy.

### 2.8 Evaluation of In Vivo Immune Activation and Immunohistochemical Effects

**Figure 8:**
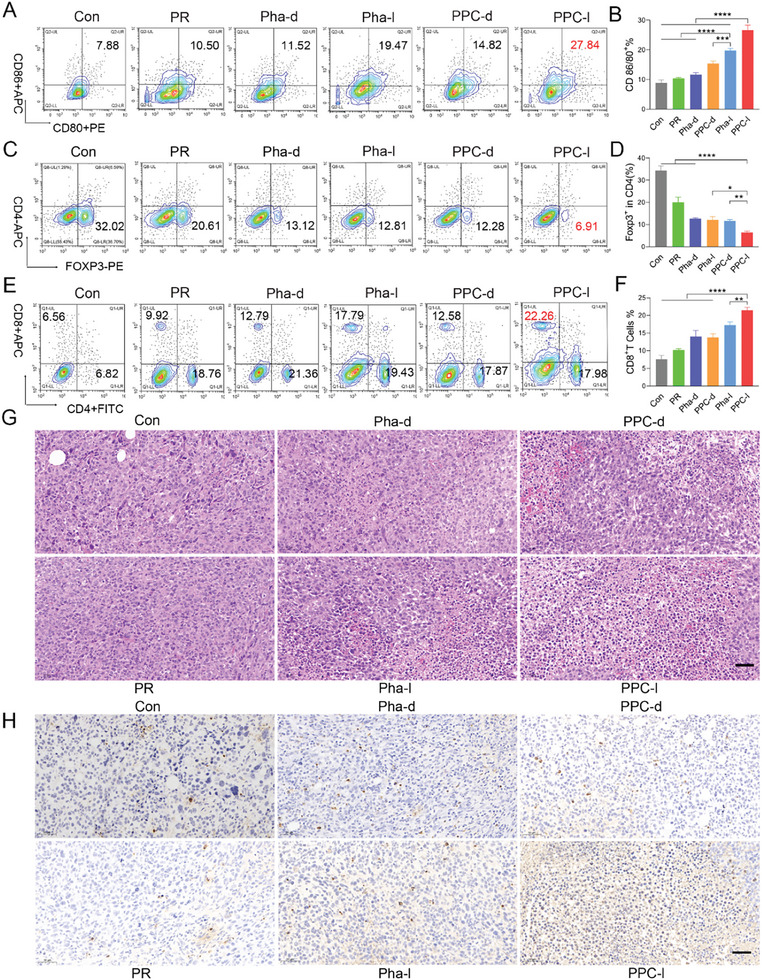
In vivo immune induction against tumors. A and B) Flow cytometry and quantitative analysis of mature DCs (CD86^+^/CD80^+^ gated) in tumor‐draining lymph nodes of mice following various treatments. Data are presented as mean ± SD (*n* = 3). C and D) Flow cytometry and quantitative analysis of Treg^+^ cells (Foxp3^+^/CD4^+^ gated) in tumors of mice following different treatments. Data are presented as mean ± SD (*n* = 3) E and F) Flow cytometry and quantitative analysis of cytotoxic T cells CD8^+^/CD4^+^ (CD8^+^/CD4^+^ gated) in tumors of mice following different treatments. Data are presented as mean ± SD (*n* = 3). G) H&E staining of tumors following different treatments, scale bar: 50 µm. H) Tunel staining of tumors following different treatments, scale bar: 50 µm. l represents light and d represents dark. Statistical significance was calculated using a one‐way analysis of variance, with ^*^ *p <* 0.05, ^**^ *p <* 0.01, ^***^ *p <* 0.001, ^****^ *p <* 0.0001.

To further investigate the immune mechanism of PPC in activating T cells, we isolated antigen‐presenting cells (APCs) (CD86^+^/CD80^+^) from tumor‐draining lymph nodes. Our findings revealed that the PPC‐light group exhibited a significantly higher proportion of DC cells, reaching 27.84%, which was 3.6 times greater than in the control group and 2.4 times higher than in the Pha‐dark group (**Figure** **8**A,B). Subsequently, we analyzed the proportions of cytotoxic CD8^+^ T cells, Treg^+^ cells, and CD4^+^ helper T cells within the tumor. These antigen‐specific T cells play a crucial role in activating adaptive immunity and directly combating cancer cells. Notably, the percentage of Treg^+^ cells decreased from 32.02% to 6.91%, representing a substantial 4.8‐fold reduction (Figure 8C,D). Furthermore, PPC‐light immunotherapy promoted the infiltration of CD8^+^ T cells (22.26%) and CD4^+^ T cells (17.98%) into the primary tumor (Figure S16B, Supporting Information), showing a 3.4‐fold and 2.6‐fold increase, respectively, compared to the control group (Figure 8E,F).

Following 5 administrations, we collected major organs from mice and conducted H&E staining, which did not reveal any significant tissue damage (Figure S17, Supporting Information). To assess the cytotoxic impact on tumors, we examined tumor tissues from mice and assessed tumor apoptosis using H&E and Tunel staining. Our results indicated minimal apoptosis in the control and PR groups, weak apoptosis in the Pha‐dark and PPC‐dark groups, and pronounced apoptotic effect in the PPC‐light group, characterized by evident nuclear shrinkage (Figure 8G,H). The PPC‐light group induced in vivo dendritic cell maturation and enriched a substantial population of cytotoxic T lymphocytes, resulting in pronounced cytotoxic effects on tumor cells.

## 3 Conclusion

In conclusion, our study introduces PPC, a pioneering peptide self‐assembly material designed for photo‐controlled immunotherapy of breast cancer. By leveraging the unique properties of Pha and HER2‐targeting peptides, PPC nanoparticles demonstrate remarkable specificity for HER2 proteins. Upon exposure to near‐infrared light, these nanoparticles generate ROS that effectively induce apoptosis and facilitate HER2 protein degradation.

Following systemic administration, PPC nanoparticles accumulate in tumor sites and undergo enzymatic hydrolysis, converting them into nanofibers within the tumor microenvironment. These nanofibers exhibit an extended retention time, significantly enhancing the ICD response. The sustained presence of nanofibers stimulates the release of key immunostimulatory molecules such as CRT and HMGB1, which activate and mature DCs in vivo. This activation boosts T‐cell infiltration and enhances the body's immune response against tumor proliferation.

However, challenges remain regarding the impact of gelatinases secreted by cancer cells during metastasis on the receptor‐mediated localization and uptake of PPC. Strategies to overcome these challenges may include optimizing drug delivery methods, such as local injections or using nanoparticle systems to improve PPC concentration at tumor sites, as well as assessing extracellular gelatinase levels to tailor PPC designs for specific tumor microenvironments.

Moreover, the potential immunogenicity of photosensitizers poses a risk of macrophage uptake limiting PPC delivery to cancer cells. While our results indicate effective HER2 protein degradation in tumor tissues, indicating minimal macrophage uptake, future applications must address this concern.

Overall, the innovative combination of PDT with peptide‐targeted nanomaterials in PPC presents a novel approach to cancer immunotherapy. By transforming “cold” tumors into “hot” tumors and initiating a robust immune response, PPC offers a promising strategy for improving therapeutic efficacy and advancing breast cancer treatment.

## 4 Experimental Section

### Synthesis of Peptides

The Pha‐PLGVRGRTYGKRPKIRT self‐assembling polypeptide was synthesized using standard solid‐phase peptide synthesis (SPPS) techniques and Fmoc‐based chemical coupling methods.^[^ ^40^, ^41^ ^]^ Briefly, the amino acids were sequentially coupled to an amino resin solid support. The resin (1 equivalence) was first swelled in anhydrous DCM for 20 min, followed by Fmoc deprotection in 20% piperidine in DMF (v/v) for 30 min. After thorough washing with DMF and DCM, the next Fmoc‐protected amino acid (2.5 equivalence), HOBT (2.5 equivalence), and DIEA (2.5 equivalence) were added for coupling. This deprotection and coupling cycle was repeated until the full peptide sequence was assembled on the resin. Finally, the Pha moiety was attached to the N‐terminus using HATU to yield the self‐assembling PPC (Pha‐PLGVRGRTYGKRPKIRT)^[^ ^38^ ^]^ polypeptide. The peptide was then cleaved from the resin using TFA for 4 h and precipitated in pre‐cooled isopropyl ether.

### Cell Culture

The TUBO cell line was obtained from the Feihui Biotechnology Co., Ltd and cultured in RPMI 1640 medium (Sangon Biotech (Shanghai) Co., Ltd.) supplemented with 2% penicillin‐streptomycin and 15% fetal bovine serum. The cells were seeded in RPMI 1640 medium in 96‐well,6‐well, or 12‐well plates and maintained at 37 °C with 5% CO_2_.

### Cytotoxicity Evaluation

TUBO cells were seeded in 96‐well plates at a density of 1 × 10^4^ cells per well, incubated for 24 h, and then treated with the drug. Different treatment groups, including PBS, Pha‐dark, Pha‐light, PPC‐dark, and PPC‐light, were established to assess the cytotoxic effects of the drugs on the cells. CCK‐8 reagent was co‐incubated with the cells and then added to the medium for 2–4 h. The absorbance at 450 nm was measured, and cell viability was evaluated. Finally, the data from each group were analyzed using GraphPad Prism 8.

### Drug Targeting

To verify the targeting of the drug, TUBO cells were seeded in a 6‐well plate at a density of 5 × 10^5^ cells per well. The cells were then treated with PPC, and the differences in cellular uptake were assessed. Cells were collected at various time points, and the internalization of PPC in TUBO cells was quantitatively analyzed by flow cytometry. The data from each group were analyzed using GraphPad Prism 8. Additionally, to confirm the ability of PPC to target the membrane protein HER2, 3 × 10^5^ cells were seeded in glass‐bottom Petri dishes, treated at 1, 4, and 8 h, and then imaged using a laser scanning confocal microscope (Leica Laser Confocal, Germany).

### The Generation of ROS

To assess the induction of ROS in TUBO cells, the ROS assay kit was employed. Cells were seeded in 6‐well plates at a density of 5 × 10^5^ cells per well and incubated for 24 h to allow for cell attachment. The experimental groups were then treated with PBS, PR, Pha‐dak, Pha‐light, PPC‐dark, or PPC‐light. The cells were stained with DCFH‐DA (Beyotime Biotechnology) according to the manufacturer's instructions. ROS production was subsequently measured using flow cytometry (Beckman Coulter, CytoFLEX).

### Full Wavelength Scanning

Pha (1 mg) and PPC (1 mg) were dissolved in 4 mL of ultrapure water containing 75% methanol. The solution was then placed in a glass dish and subjected to full wavelength scanning using an ultraviolet spectrophotometer to determine the locations of the major absorption peaks of the compounds (Thermo, Biomate 160).

### Analysis by Electron Microscopy

PR (3 mg) and PPC (3 mg) were dissolved in 3 mL of ultrapure water and continuously stirred for 4 h. Subsequently, 300 µL of the solution was applied to a copper mesh grid, incubated for 24 h, and then stained with uranyl acetate. After 12 h, the samples were imaged using a transmission electron microscope (JEM‐2100 TEM, Japan). Additionally, 3 mg of PR and 3 mg of PPC were separately affixed to the conductive adhesive and mounted on a silicon wafer. The samples were then sputter‐coated with gold and imaged using a scanning electron microscope (ZEISS Sigma 300, Germany).

In a separate experiment, 6 × 10^5^ TUBO cells were seeded in culture dishes and treated with 0.5 µm PPC for 4 h. The cells were then harvested using a cell scraper, fixed with a specialized electron microscopy fixative (2% glutaraldehyde), stained with uranyl acetate, and imaged using a biological transmission electron microscope (Hitachi HT7800, Japan).

### Apoptosis Assessment

In brief, TUBO cells were seeded at a density of 5 × 10^5^ cells per well in a 6‐well plate and cultured for 24 h. Upon reaching approximately80% confluence, the experimental groups were treated with PBS, PR, Pha‐dak, Pha‐light, PPC‐dark, or PPC‐light for an additional 24 h. The cells were then harvested, stained with V‐FITC and propidium iodide (PI) according to the manufacturer's protocol, and analyzed by flow cytometry. Finally, the data from each group were analyzed using GraphPad Prism 8. Additionally, TUBO cells were cultured in a glass dish, and cellular apoptosis was visualized under a microscope 24 h post‐administration.

### Protein Expression Analysis

To assess HER2 protein expression in TUBO cells, Western blotting was employed to measure the levels of HER2 expression. TUBO cells were seeded in 6‐well plates at a density of 6 × 10^5^ cells per well. Following a 24 h incubation period, the experimental group was divided into treatment subgroups including PBS, PR, Pha‐dak, Pha‐light, PPC‐dark, and PPC‐light. After 24 h of drug treatment, cell membrane proteins were extracted and analyzed to evaluate HER2 protein degradation.

### Immune induction Experiment In Vitro

TUBO cells were seeded into 6‐well plates at a density of 6 × 10^5^ cells and cultured in the medium for 24 h. Subsequently, various agents including PBS, PR, Pha‐dak, Pha‐light, PPC‐dark, and PPC‐light were introduced to the medium. After a 24 h incubation period, the supernatant was collected for ELISA analysis of ATP and HMGB‐1. Separately, 3 × 10^5^ TUBO cells were seeded on a glass substrate and treated with CRT antibody, followed by staining with Alexa Fluor 594 secondary antibody (Abbkine Scientific Co., Ltd.). The cellular expression of drug‐induced CRT was visualized using a confocal laser microscope (Olympus, fv3000).

### In the In Vitro Experiment Aimed at Inducing DC Maturation

TUBO cells and RAW264.7 cells were initially cultured in 6‐well plates at a seeding density of 4 × 10^5^ cells. After 24 h in standard medium, the cells were subjected to different treatment groups, including PBS, PR, Pha‐dak, Pha‐light, PPC‐dark, and PPC‐light. Following another 24 h incubation, the cells were harvested, stained with *CD*86^+^/*CD*80^+^ flow‐antibody (Becton, Dickinson and Company), washed with PBS, and analyzed using flow cytometry. Furthermore, RAW264.7 cells were cultured in glass dishes. After 24 h of treatment, a high‐resolution fiberscope was employed to observe the induced maturation of DCs.

### In Vivo Immune Factor Induction Experiment

The breast cancer animal model was established using 8‐week‐old female *Balb*/*c* mice weighing approximately20 g, sourced from the animal laboratory at the School of Translational Medicine, Shanghai University. Following the establishment of the tumor model, mice received 5 doses of drug treatment. Subsequently, peripheral blood was collected, and centrifuged at 4000 rpm, and the supernatant was analyzed for the cytokines TNF−α, IL‐6, and IFN−γ. ELISA assays were performed in strict accordance with the manufacturer's instructions (MultiSciences Biotech Co., Ltd.).

### Animal Experiment Operation and Immune Stimulation In Vivo

In this study, a breast cancer murine model was employed using 8‐week‐old female Balb/c mice, each weighing approximately20 g, sourced from the School of Translational Medicine's animal laboratory at Shanghai University. To create a tumor model, 3 × 10^5^ TUBO cells were inoculated into the right hind limb of each mouse. The experimental design included a control group receiving saline and various treatment groups (PR, Pha‐dark, Pha‐light, PPC‐dark, PPC‐light). Drug administration commenced once tumors reached a volume of 60 mm^3^, with treatments applied bi‐daily using a 660 nm laser (250 mm cm^−2^ power density, 6 min exposure, and total light dose of 90 J cm^−2^). Mice were monitored bi‐daily for changes in weight and tumor volume. Following 5 treatment cycles, mice were euthanized via cervical dislocation. Organs such as the heart, liver, spleen, lungs, and kidneys were harvested for H&E staining to evaluate potential drug‐induced damage. Tumor specimens underwent HER2 immune fluorescence staining, H&E staining, and Tunel immune histochemistry to assess the therapeutic effects. To investigate T cell‐mediated immune responses in vivo, tumor and lymphoid tissues were extracted and processed under sterile conditions with ≈900 mg of material. Tissues were digested with Type II collagenase at 37 °C for 50 min, then strained through a 75 µm cell strainer to remove aggregates. Following PBS washes, single‐cell suspensions were prepared, and lymphocytes were isolated using a TBD LTS1092PK mouse splenic lymphocyte isolation kit. After additional filtering to remove debris, cells were suspended in PBS, adjusting the concentration to 1.5 × 10^5^ cells in 300 µL. Lymphocytes were labeled with CD80^+^/CD86^+^ primary antibodies, while tumor cells were stained with antibodies against CD8^+^/CD4^+^ and Foxp3^+^/CD4^+^. Following staining, cells were incubated at room temperature for 25 min, washed, and prepared for flow cytometry analysis using a Beckman Coulter CytoFLEX system.

## Conflict of Interest

The authors declare no conflict of interest.

## Author Contributions

C.G. and F.G. synthesized the compounds and completed most biological assays, G.W. and J.L. performed the literature search and data collection. C.S., S.H., and H.H. performed data analysis and contributed to the writing–review, and editing of the manuscript. All authors have approved the final version of the manuscript.

## Supporting information



Supporting Information

## Data Availability

The data that support the findings of this study are available from the corresponding author upon reasonable request.
