# Supplementary material for: Precise HER2 Protein Degradation via Peptide‐Conjugated Photodynamic Therapy for Enhanced Breast Cancer Immunotherapy
Source: Adv Sci (Weinh). 2024 Nov 18;12(2):2410778. doi: 10.1002/advs.202410778 (PMC11727380; doi:10.1002/advs.202410778)
Supplement: Supplementary file 1 — Supporting Information [file ADVS-12-2410778-s001.docx]

Supporting Information

Precise HER2 Protein Degradation via Peptide-Conjugated Photodynamic Therapy for Enhanced Breast Cancer Immunotherapy

Changyong Guo,^a,†^ Fei Gao, ^a,†^ Guoyuan Wu ^a^, Jinqiu Li ^a^, Chunquan Sheng^b,*^ Shipeng He ^a,*^,and Honggang Hu^a,*^

C.Y. Guo, F. Gao, G.Y. Wu, J.Q. Li, S.P. He, and H.G. Hu

^a^ School of Medicine or Institute of Translational Medicine, Shanghai Engineering Research Center of Organ Repair, Shanghai University, 99 Shangda Road, Shanghai 200444, P.R. China.

E-mail: [heshipeng@shu.edu.cn](mailto:heshipeng@shu.edu.cn); hhu66@shu.edu.cn.

C.Q. Sheng

^b^ The Center for Basic Research and Innovation of Medicine and Pharmacy (MOE), School of Pharmacy, Second Military Medical University (Naval Medical University), 325 Guohe Road, Shanghai 200433, P.R. China

E-mail:[shengcq@smmu.edu.cn](mailto:shengcq@smmu.edu.cn)

**Table of contents**

[Figure S1 The chemical structure formulas of PR and PPC. S3](#_Toc165831149)

[Figure S2 Morphological Characterization. S3](#_Toc165831150)

[Figure S3 Morphological characterization of PPC before and after enzymatic hydrolysis S4](#_Toc165831151)

[Figure S4 HPLC Characterization of PPC and Pha-PLG S4](#_Toc165831152)

[Figure S5 The mass spectrum of Pha-PLG S5](#_Toc165831153)

[Figure S6 Results of flow cytometry testing different cell affinities S5](#_Toc165831153)

[Figure](#_Toc165831157) S7 Fluorescence quantification analysis of HER2 protein degradation. S6

[Figure S8 Verification of *in vitro* protein degradation experiment results. S6](#_Toc165831155)

[Figure](#_Toc165831153) S9 Results of the effect of PPC on ER protein expression. S7

[Figure S10 Whole-membrane protein results. S7](#_Toc165831157)

[Figure S11 Representative visual micrograph depicting apoptosis. S8](#_Toc165831155)

[Figure S12 Statistical analysis of immune induction experiments in vitro. S8](#_Toc165831158)

[Figure](#_Toc165831154) S13 llustrate typical images of in vitro-induced maturation of dendritic cells. S9

[Figure S14 The animal infrared imaging results of PPC. S9](#_Toc165831156)

[Figure S15 Immunohistochemical Analysis. S10](#_Toc165831160)

[Figure](#_Toc165831159) S16 Body weight of mice and in vivo immune T cell testing outcomes. S10

[Figure S17 Histological evaluation. S11](#_Toc165831161)

[Materials and methods. S11](#_Toc165831161)

Spectra[. S17](#_Toc165831161)

References[. S19](#_Toc165831161)


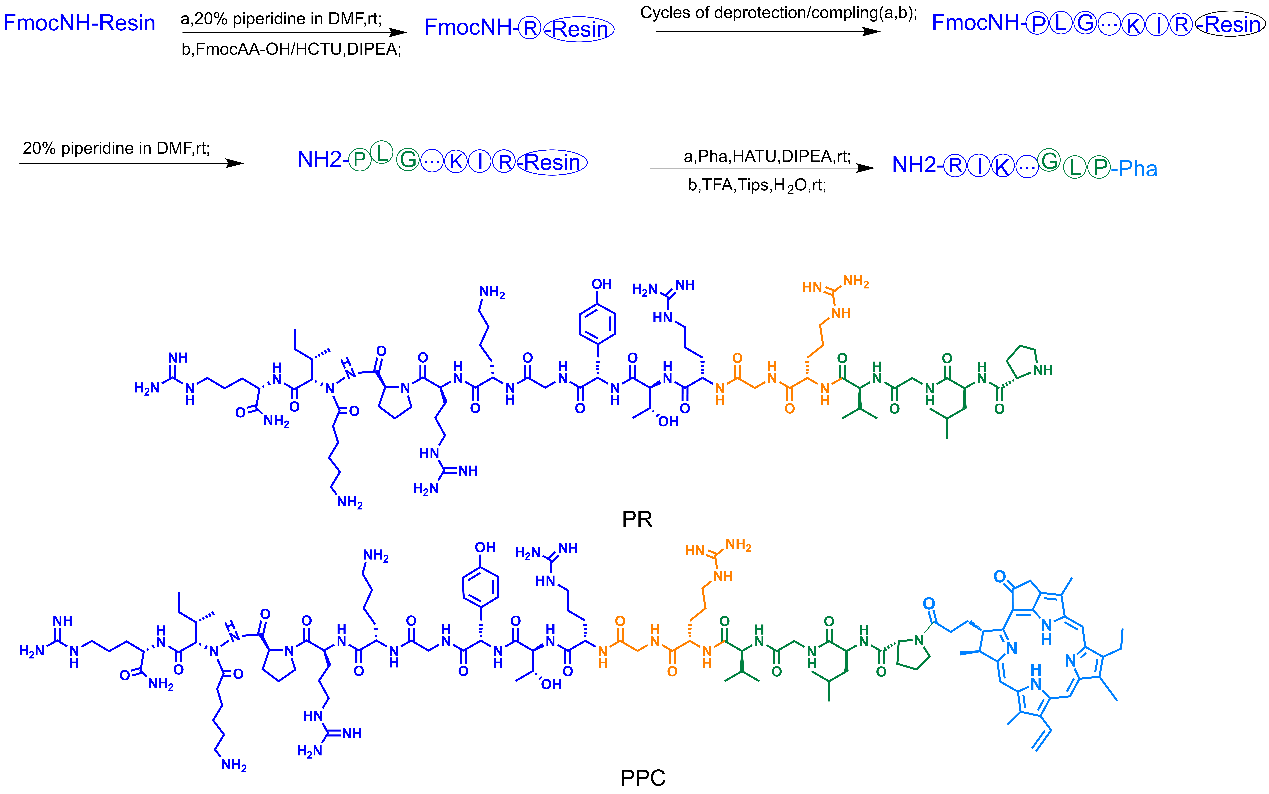


**Figure S1** The chemical structure formulas of PR and PPC are presented.


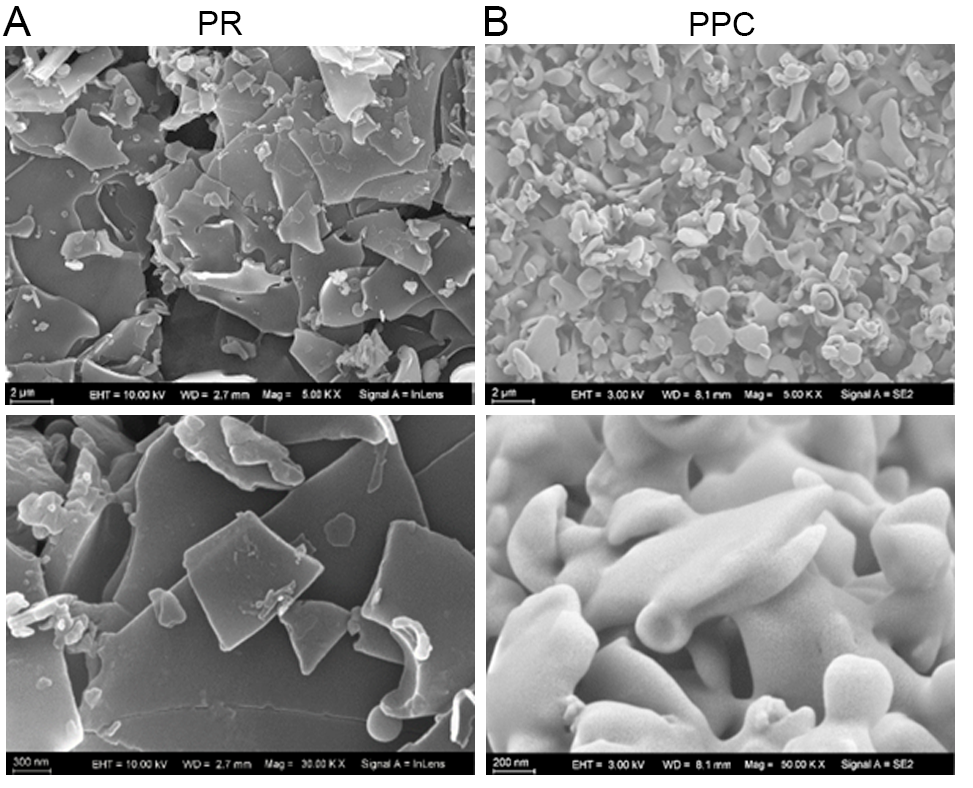


Figure S2 Morphological Characterization. (A) SEM analysis of PR in the absence of water. (B) SEM analysis of PPC under anhydrous conditions.


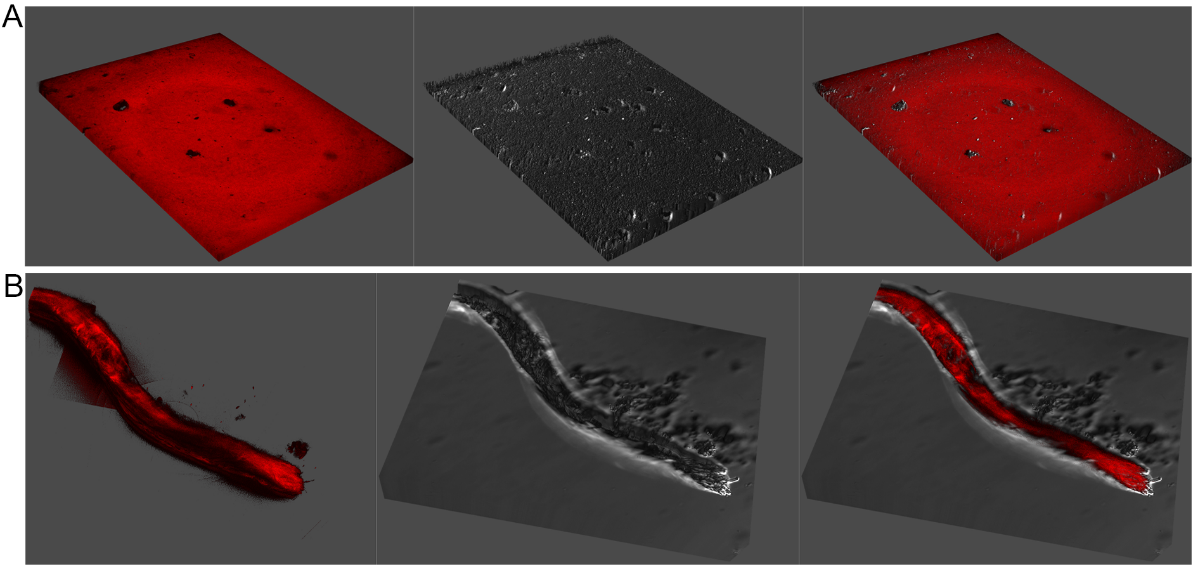


Figure S3 Morphological characterization of PPC before and after enzymatic hydrolysis using ultra-high resolution confocal microscopy. (A) PPC exhibits a spherical morphology in ultra-pure water. (B) Subsequent enzymatic hydrolysis induces a transition to a nanofibrous structure.


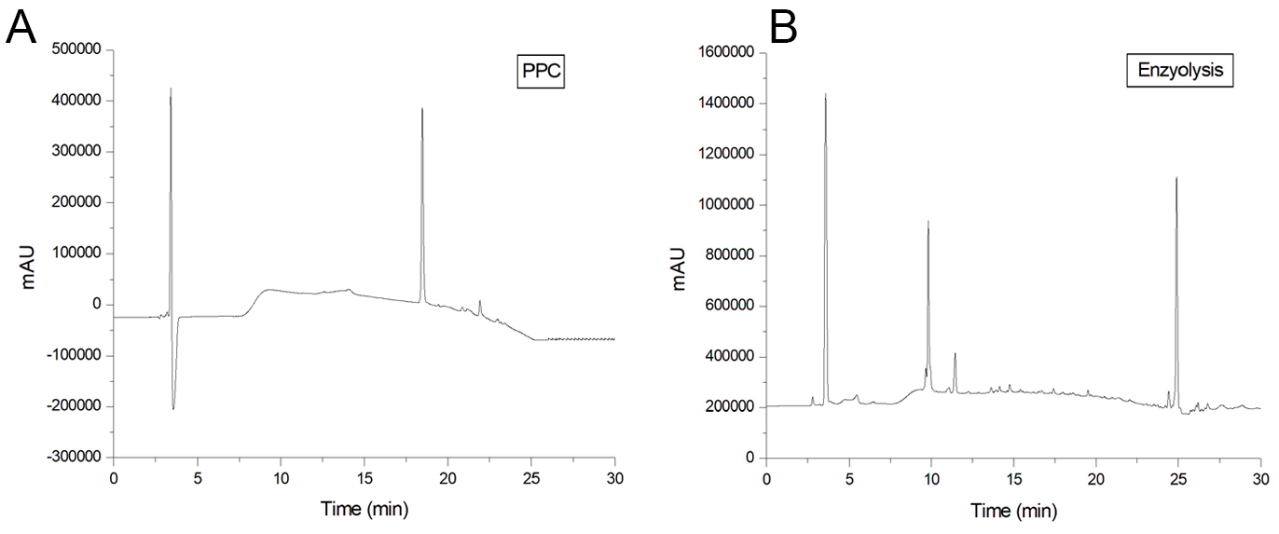


Figure S4 HPLC Characterization (A) The HPLC retention time of the PPC compound. (B) The HPLC profile of PPC after *in vitro* cleavage by gelatinase.


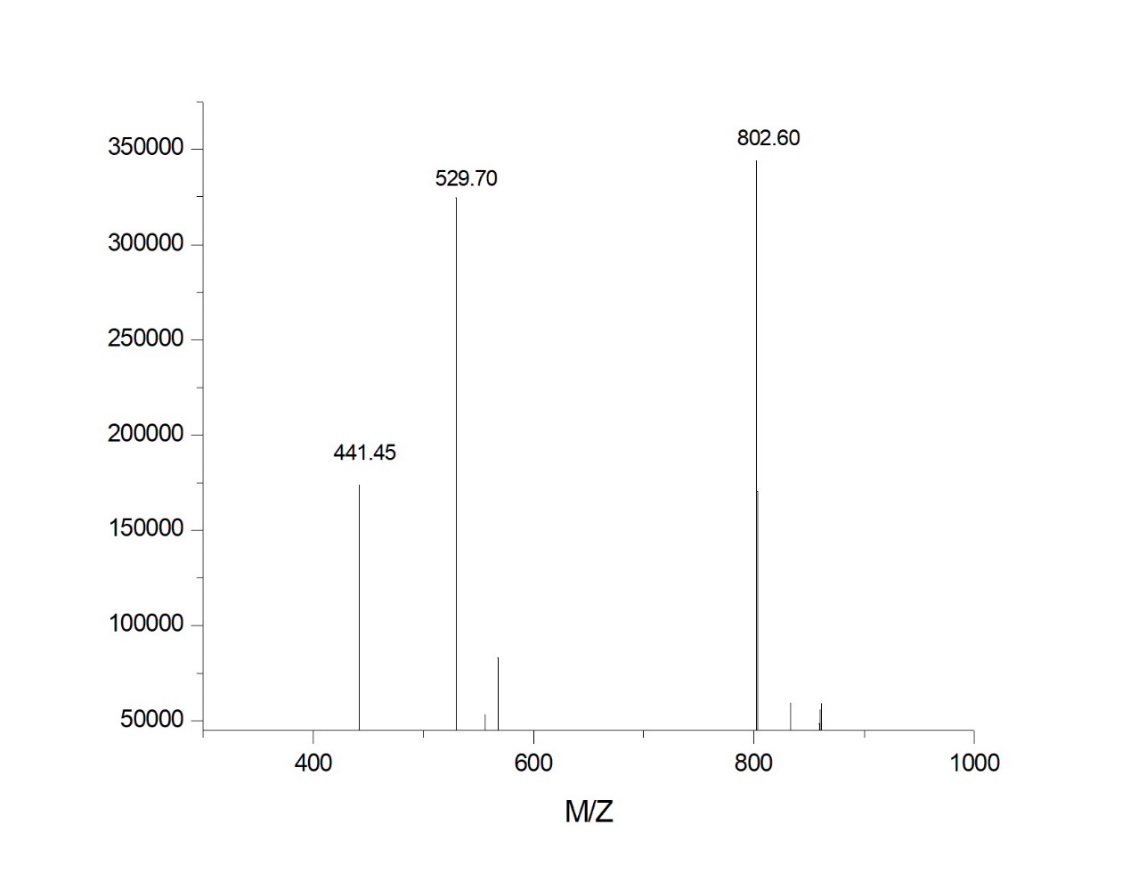


Figure S5 The mass spectrum of PPC was revealed following enzymatic hydrolysis by gelatinase. Specifically, residual fragments of Pha-PLG were detected after PPC cleavage.


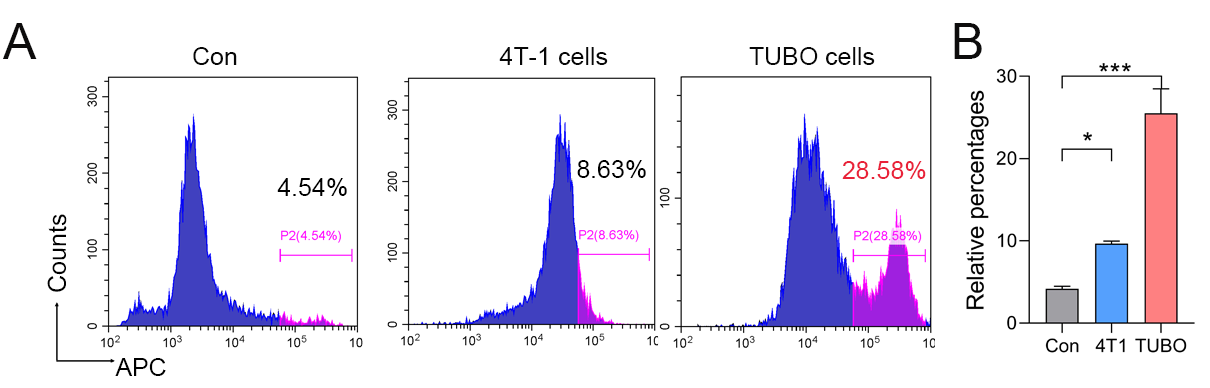


Figure S6 (A) Results of flow cytometry testing different cell affinities. (B) Statistical results of different cell affinities. Data are presented as mean ± SD (n = 3) Statistical significance was calculated using a one-way analysis of variance. **p < 0.01, ***p < 0.001, ****p < 0.0001.


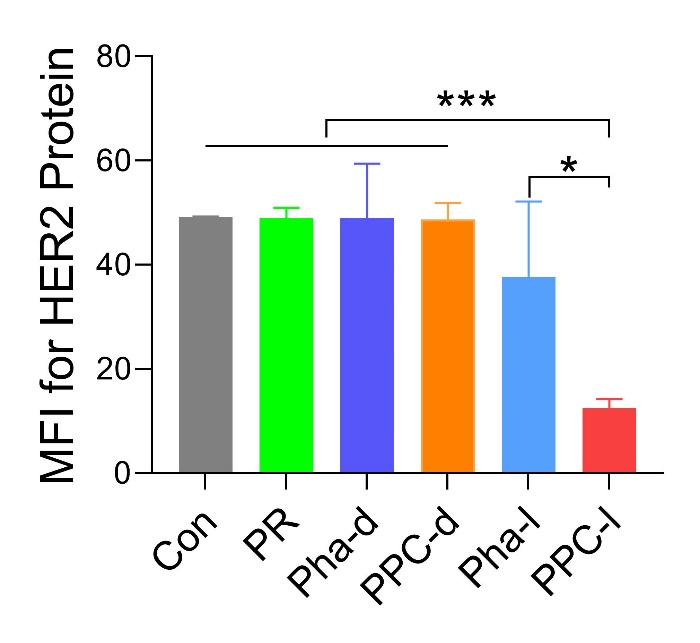


**Figure S7** Fluorescence quantification analysis of HER2 protein degradation in different groups. l represents light and d represents dark. Data are presented as mean ± SD (n = 3). Statistical significance was calculated using a one-way analysis of variance. **p < 0.01, ***p < 0.001, ****p < 0.0001.


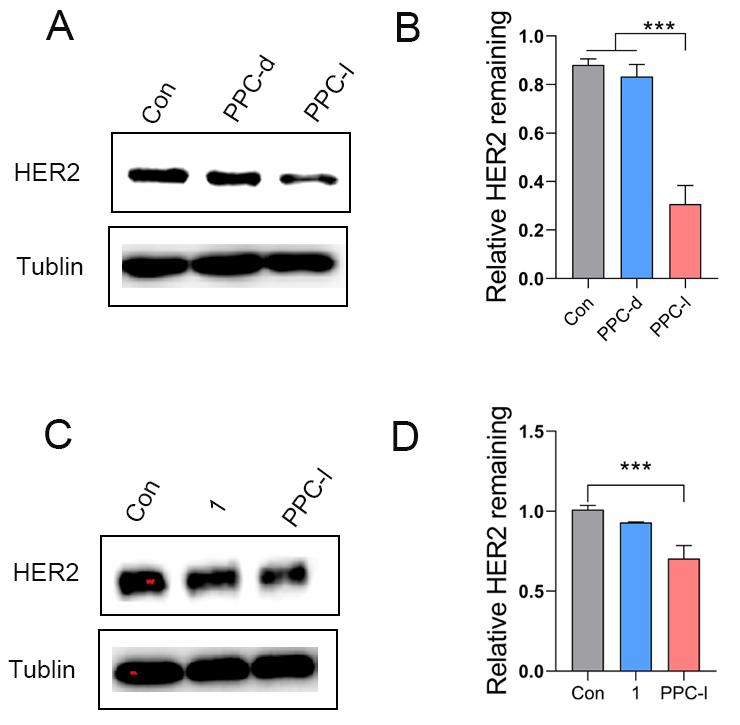


**Figure S8** Verification of *in vitro* protein degradation experiment results. (A) Verification of *in vitro* protein degradation experiment results by Western blot analysis, with a concentration of 0.5 μM for the PPC-light group. (B) Statistical results of protein degradation. Data are presented as mean ± SD (n = 3). (C) Verification of *in vitro* protein degradation experiment results. 1 indicates the ROS scavenger and 0.25 μM PPC-light. (D) Statistical results of protein degradation. l represents light and d represents dark. Data are presented as mean ± SD (n = 3). Statistical significance was calculated using a one-way analysis of variance. ***p < 0.001.


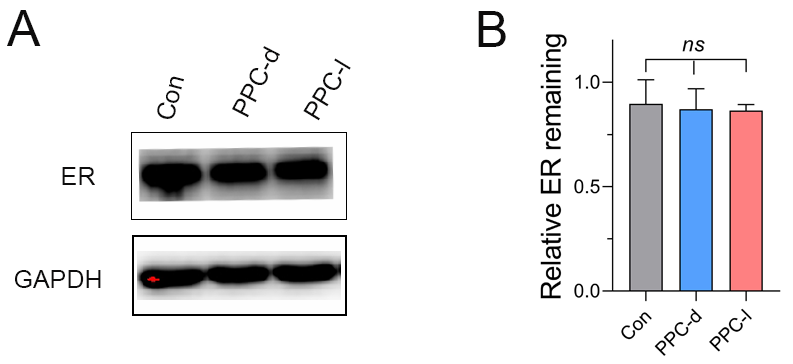


**Figure S9** Results of the effect of PPC on ER protein expression. (A) Western blot analysis verifies the PPC effect on ER protein expression. (B) Statistical results of PPC effect on ER protein expression analysis. l represents light and d represents dark.

Data are presented as mean ± SD (n = 3). Statistical significance was calculated using a one-way analysis of variance. ns, not significant.


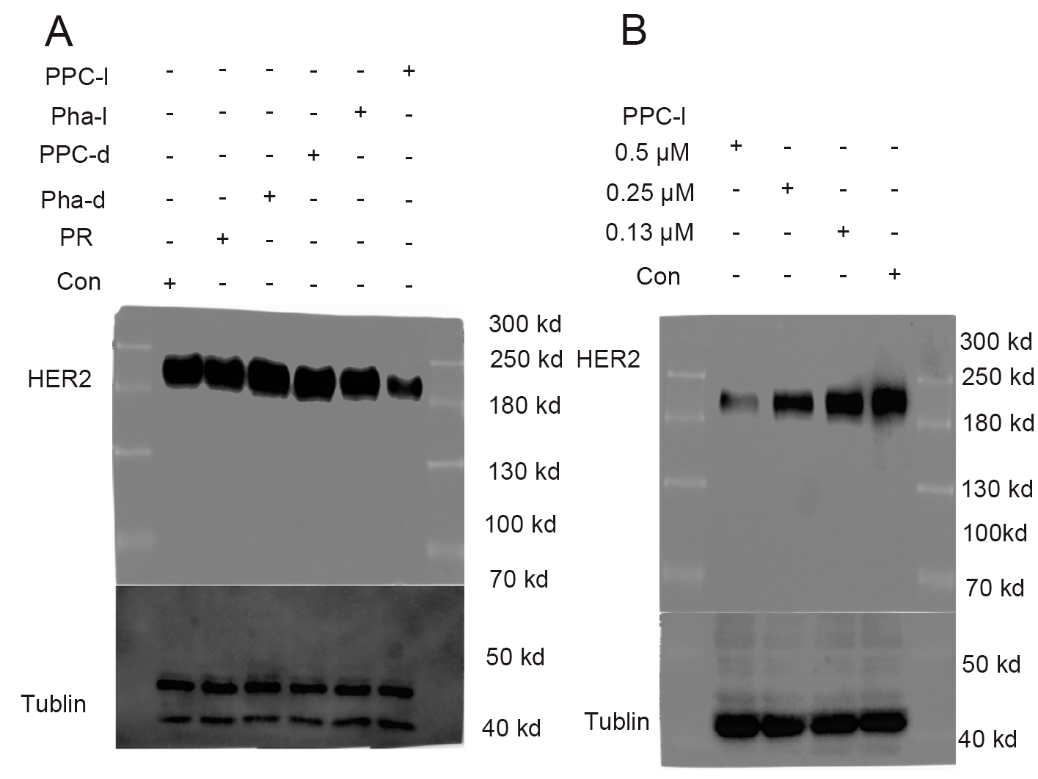


**Figure S10** Whole-membrane protein results. (A) Protein expression results in different groups after drug therapy. (B) Protein expression results after PPC drug therapy at different concentrations. l represents light and d represents dark.


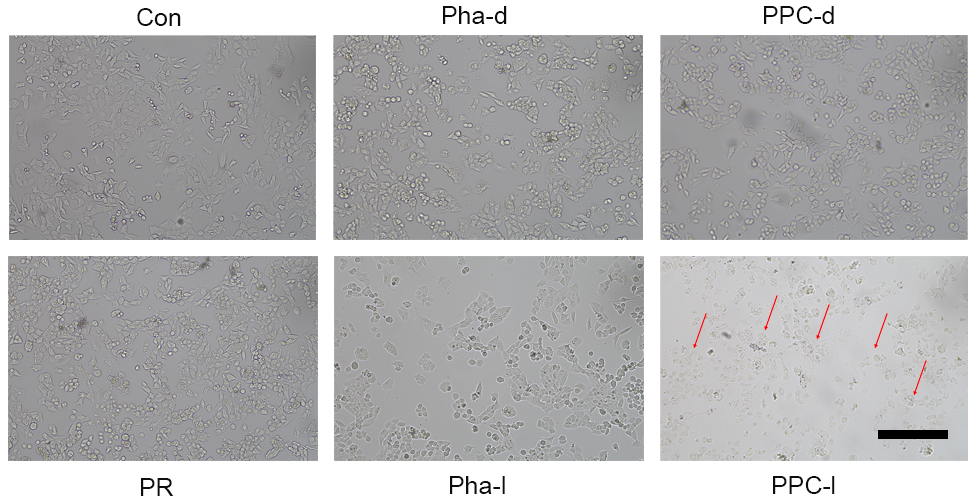


**Figure S11** Representative visual micrograph depicting apoptosis. l represents light and d represents dark, scale bars, 200 μm.


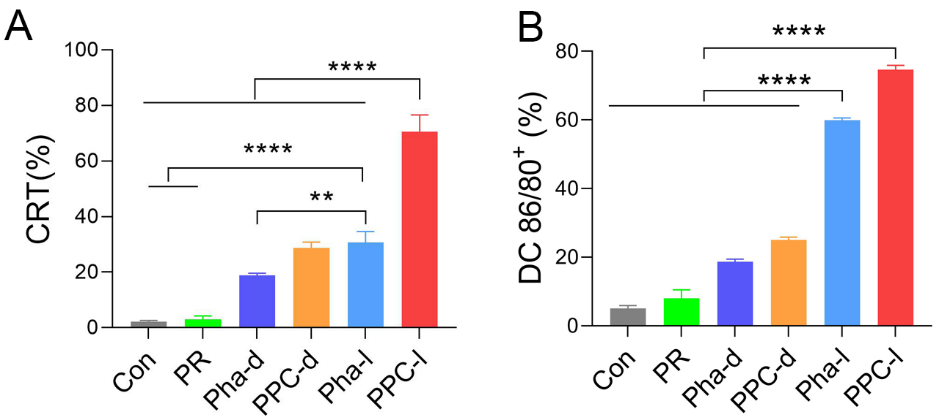


Figure S12 Statistical analysis of immune induction experiments *in vitro*. (A) Flow cytometric evaluation of CRT induction. Data are presented as mean ± SD (n = 3). (B) *In vitro* assessment of DCs maturation induction. Data are presented as mean ± SD (n = 3). l represents light and d represents dark. Statistical significance was calculated using a one-way analysis of variance. **p < 0.01, ***p < 0.001, ****p < 0.0001.


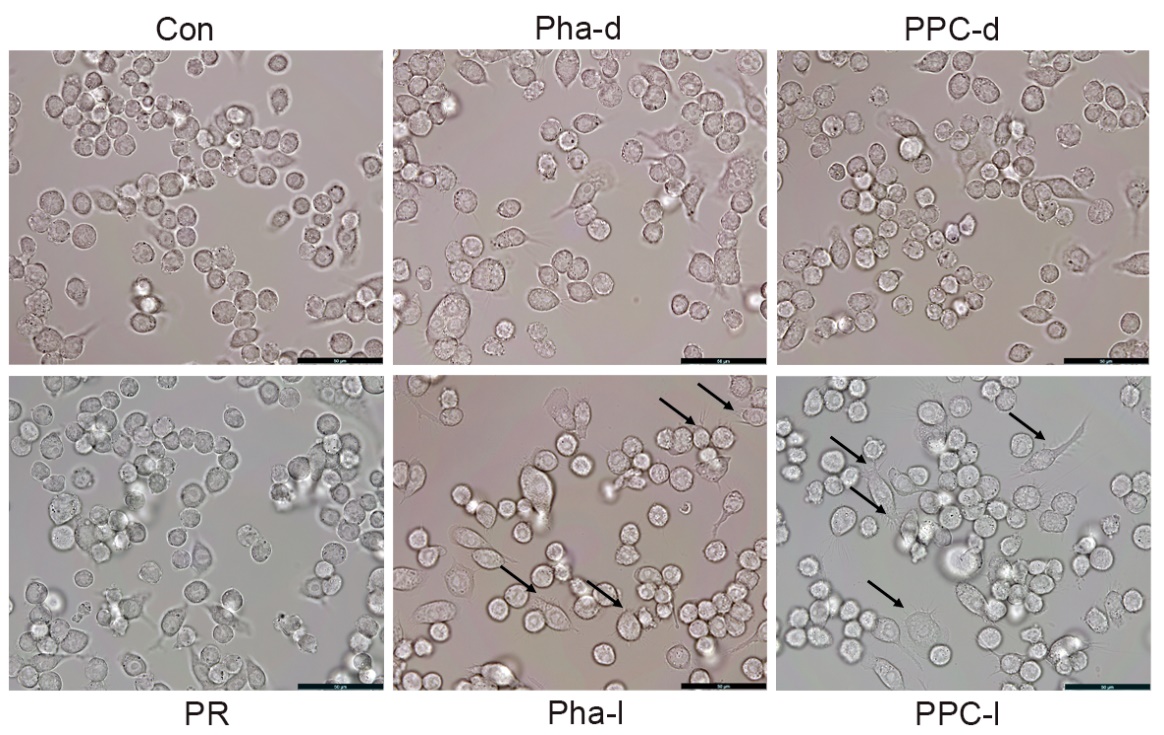


Figure S13 llustrate typical images of *in vitro*-induced maturation of dendritic cells. l represents light and d represents dark, scale bars, 50 μm.


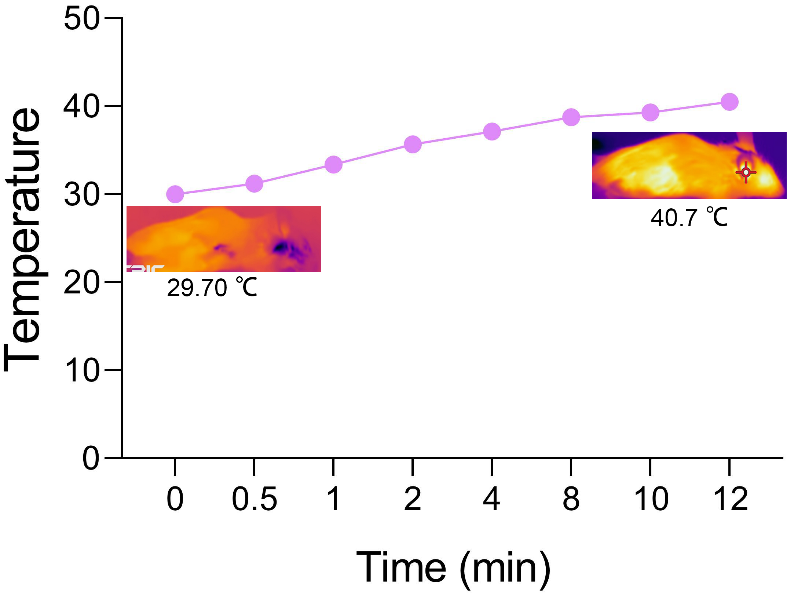


**Figure S14** The animal infrared imaging results of PPC.

*
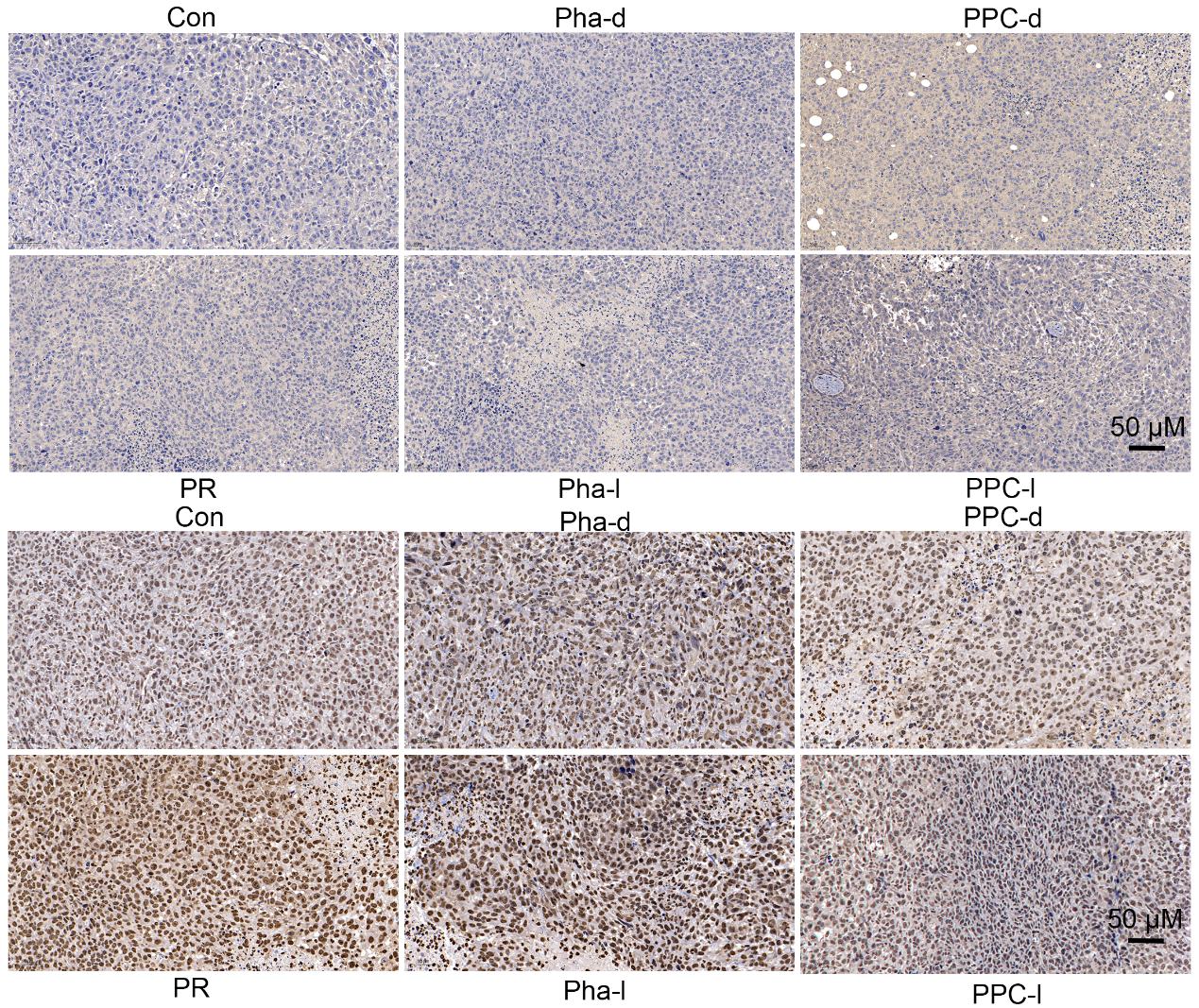
*

**Figure S15** Immunohistochemical Analysis. (A) CRT immunohistochemical staining in tumor tissues after 3 doses of treatment. (B) HMGB1 immunohistochemical staining in tumor tissues after 3 doses of treatment, scale bars, 50 μm. l represents light and d represents dark.


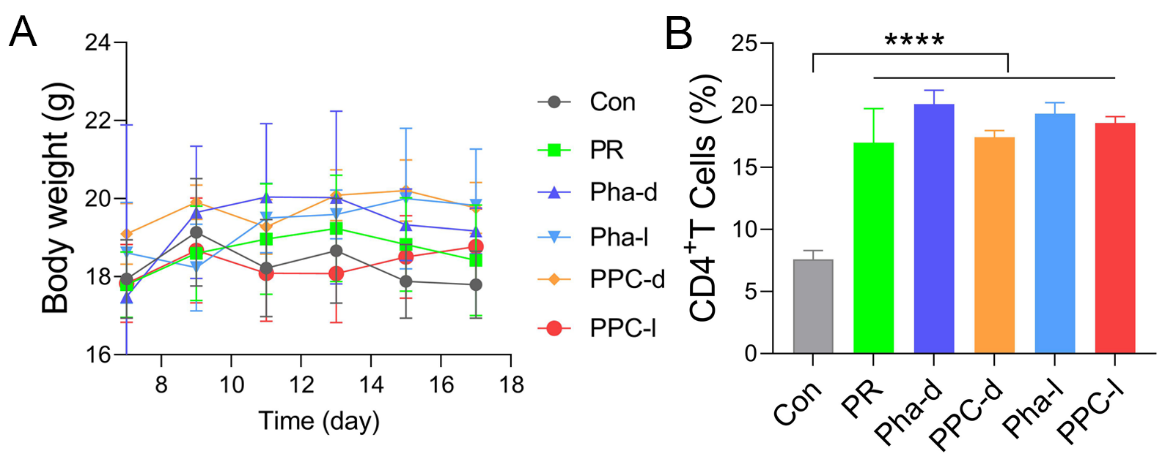


Figure S16 Present findings on *in vivo* immune T cell responses. (A) Body weight change curve in each group. Data are presented as mean ± SD (n = 3). (B) *In vivo* CD4^+^ T cell profiling in tumor microenvironment. Data are presented as mean ± SD (n = 3). l represents light and d represents dark. The p values were calculated by ANOVA with Tukey’s test. **p < 0.01, ***p < 0.001, ****P < 0.0001.


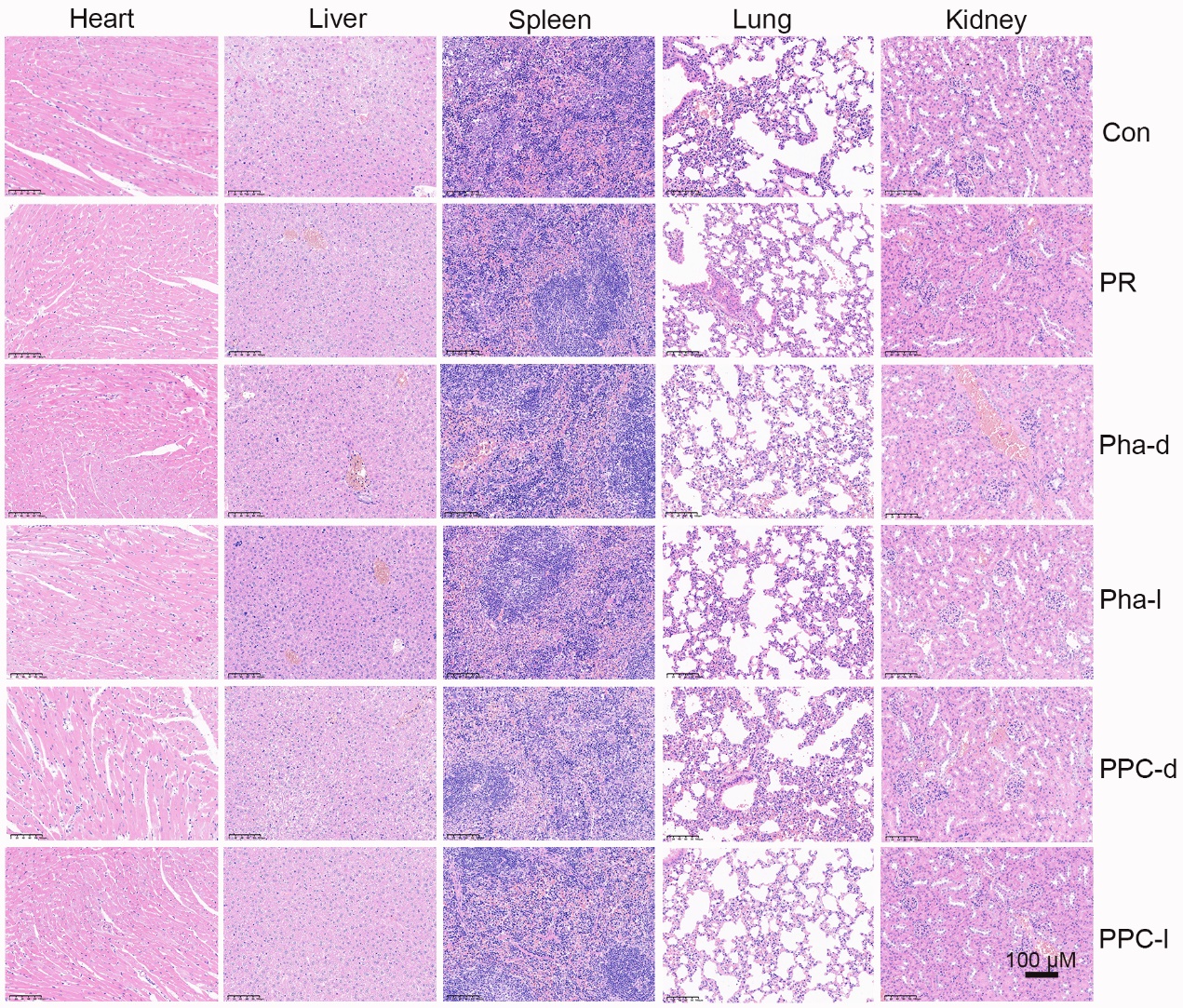


Figure S17 Histological evaluation. H&E-stained sections of the heart, liver, spleen, lung, and kidney from mice after different drug administration regimens are presented. l represents light and d represents dark, scale bars,100 μm.

**Materials and Methods**

*General Methods:* Methanol (MeOH), dichloromethane (DCM), *N*, *N*-dimethylformamide (DMF), *N*, *N*-diisopropylethylamine (DIEA), *N*, *N'*-diisopropylcarbodiimide (DIC), 1-hydroxybenzotriazole (HOBT), trifluoroacetic acid, formic acid, dialysis bags were purchased from Shanghai Titan Scientific Co., Ltd. Trifluoroacetic acid(TFA) was purchased from Aladdin Biochemical Technology Co., Ltd. Protein immunoblotting secondary antibodies, reactive oxygen species assay kit, western blot primary and secondary antibody dilution solutions, nuclear-staining reagent (DAPI), CCK-8 assay kit and Chemiluminescent Assay Kit were purchased from Beyotime Biotechnology. 1640 medium, trypsin, penicillin-streptomycin, and chemiluminescent secondary antibodies were purchased from Sangon Biotech (Shanghai) Co., Ltd. HER2 mouse primary antibody was purchased from Cell Signaling Technology. Protein mark, CD8^+^, CD4^+^, CD86^+^, CD80^+^, and Foxp3^+^ flow cytometry antibodies were purchased from Becton, Dickinson, and Company. TNFα, IL-6, IFN-γ ELISA kits, and cell apoptosis assay kit were purchased from MultiSciences Biotech Co., Ltd. Electrophoresis buffer, and transfer buffer were purchased from Epizyme Biomedical Technology Co., L. Serum was purchased from Gibco.

**Experimental section**

*Synthesis of peptides:* The Pha-PLGVRGRTYGKRPKIRT self-assembling polypeptide was synthesized using standard solid-phase peptide synthesis (SPPS) techniques and Fmoc-based chemical coupling methods^[1]^. Briefly, the amino acids were sequentially coupled to an amino resin solid support. The resin (1 equivalence) was first swelled in anhydrous DCM for 20 min, followed by Fmoc deprotection in 20% piperidine in DMF (v/v) for 30 min. After thorough washing with DMF and DCM, the next Fmoc-protected amino acid (2.5 equivalence), HOBT (2.5 equivalence), and DIEA (2.5 equivalence) were added for coupling. This deprotection and coupling cycle was repeated until the full peptide sequence was assembled on the resin. Finally, the Pha moiety was attached to the N-terminus using HATU to yield the self-assembling PPC (Pha-PLGVRGRTYGKRPKIRT)^[2]^ polypeptide. The peptide was then cleaved from the resin using TFA for 4 h and precipitated in pre-cooled isopropyl ether.

*Cell culture:* The TUBO cell line was obtained from the Beijing Feihui Biotechnology Co., Ltd and cultured in 1640 medium (Sangon Biotech Co., Ltd Shanghai) supplemented with 2% penicillin-streptomycin and 15% fetal bovine serum. The cells were seeded in 1640 medium in 96-well,6-well, or 12-well plates and maintained at 37°C with 5% CO_2_.

*Cytotoxicity evaluation:* TUBO cells were seeded in 96-well plates at a density of 1×10^4^ cells per well, incubated for 24 h, and then treated with the drug. Different treatment groups, including PBS, Pha-dark, Pha-light, PPC-dark, and PPC-light, were established to assess the cytotoxic effects of the drugs on the cells. CCK-8 reagent was co-incubated with the cells and then added to the medium for 2-4 h. The absorbance at 450 nm was measured, and cell viability was evaluated. Finally, the data from each group were analyzed using GraphPad Prism 8.

*Drug targeting*: To verify the targeting of the drug, TUBO cells were seeded in a 6-well plate at a density of 5×10^5^ cells per well. The cells were then treated with PPC, and the differences in cellular uptake were assessed. Cells were collected at various time points, and the internalization of PPC in TUBO cells was quantitatively analyzed by flow cytometry. The data from each group were analyzed using GraphPad Prism 8. Additionally, to confirm the ability of PPC to target the membrane protein HER2, 3×10^5^ cells were seeded in glass-bottom Petri dishes, treated at 1 h, 4 h, and 8 h, and then imaged using a laser scanning confocal microscope (Leica Laser Confocal, Germany).

*The generation of ROS:* To assess the induction of reactive oxygen species (ROS) in TUBO cells, the ROS assay kit was employed. Cells were seeded in 6-well plates at a density of 5×10^5^ cells per well and incubated for 24 h to allow for cell attachment. The experimental groups were then treated with PBS, PR, Pha-dak, Pha-light, PPC-dark, or PPC-light. The cells were stained with DCFH-DA (Beyotime Biotechnology) according to the manufacturer's instructions. ROS production was subsequently measured using flow cytometry (Beckman Coulter, CytoFLEX).

*Full wavelength scanning:* 1 mg of Pha and 1 mg of PPC were dissolved in 4 mL of ultrapure water containing 75% methanol. The solution was then placed in a glass dish and subjected to full wavelength scanning using an ultraviolet spectrophotometer to determine the locations of the major absorption peaks of the compounds (Thermo, Biomate 160).

*Analysis by electron microscopy:* 3 mg of PR and PPC were dissolved in 3 mL of ultrapure water and continuously stirred for 4 h. Subsequently, 300 μL of the solution was applied to a copper mesh grid, incubated for 24 h, and then stained with uranyl acetate. After 12 h, the samples were imaged using a transmission electron microscope (JEM-2100 TEM, Japan). Additionally, 3 mg of PR and 3 mg of PPC were separately affixed to the conductive adhesive and mounted on a silicon wafer. The samples were then sputter-coated with gold and imaged using a scanning electron microscope (ZEISS Sigma 300, Germany).

In a separate experiment, 6×10^5^ TUBO cells were seeded in culture dishes and treated with 0.5 μM PPC for 4 h. The cells were then harvested using a cell scraper, fixed with a specialized bio-electron microscopy fixative (2% glutaraldehyde), stained with uranyl acetate, and imaged using a biological transmission electron microscope (Hitachi HT7800, Japan).

*Apoptosis assessment:* In brief, TUBO cells were seeded at a density of 5×10^5^ cells per well in a 6-well plate and cultured for 24 h. Upon reaching approximately 80% confluence, the experimental groups were treated with PBS, PR, Pha-dak, Pha-light, PPC-dark, or PPC-light for an additional 24 h. The cells were then harvested, stained with V-FITC and propidium iodide (PI) according to the manufacturer's protocol, and analyzed by flow cytometry. Finally, the data from each group were analyzed using GraphPad Prism 8. Additionally, TUBO cells were cultured in a glass dish, and cellular apoptosis was visualized under a microscope 24 h post-administration.

*Protein expression analysis:* To assess HER2 protein expression in TUBO cells, Western blotting was employed to measure the levels of HER2 expression. TUBO cells were seeded in 6-well plates at a density of 6×10^5^ cells per well. Following a 24 h incubation period, the experimental group was divided into treatment subgroups including PBS, PR, Pha-dak, Pha-light, PPC-dark, and PPC-light. After 24 h of drug treatment, cell membrane proteins were extracted and analyzed to evaluate HER2 protein degradation.

*Immune induction experiment in vitro:* TUBO cells were seeded into 6-well plates at a density of 6×10^5^ cells and cultured in the medium for 24 h. Subsequently, various agents including PBS, PR, Pha-dak, Pha-light, PPC-dark, and PPC-light were introduced to the medium. After a 24 h incubation period, the supernatant was collected for ELISA analysis of ATP and HMGB-1. Separately, 3×10^5^ TUBO cells were seeded on a glass substrate and treated with calretin (CRT) antibody, followed by staining with Alexa Fluor 594 secondary antibody (Beyotime Biotechnology Co., Ltd.). The cellular expression of drug-induced CRT was visualized using a confocal laser microscope (Olympus, fv3000).

*In the in vitro experiment aimed at inducing DC maturation:* TUBO cells and RAW264.7 cells were initially cultured in 6-well plates at a seeding density of 4×10^5^ cells. After 24 h in standard medium, the cells were subjected to different treatment groups, including PBS, PR, Pha-dak, Pha-light, PPC-dark, and PPC-light. Following another 24 h incubation, the cells were harvested, stained with *CD*86^+^/*CD*80^+^ flow-antibody

(Becton, Dickinson and Company), washed with PBS, and analyzed using flow cytometry. Furthermore, RAW264.7 cells were cultured in glass dishes. After 24 h of treatment, a high-resolution fiberscope was employed to observe the induced maturation of DCs.

*In vivo immune factor induction experiment:* The breast cancer animal model was established using 8-week-old female *Balb*/*c* mice weighing approximately 20 g, sourced from the animal laboratory at the School of Translational Medicine, Shanghai University. Following the establishment of the tumor model, mice received 5 doses of drug treatment. Subsequently, peripheral blood was collected, and centrifuged at 4000 rpm, and the supernatant was analyzed for the cytokines TNF−α, IL-6, and

IFN−γ. ELISA assays were performed in strict accordance with the manufacturer’s instructions (MultiSciences Biotech Co., Ltd.).

*Animal experiment operation and Immune stimulation in vivo:* In this study, we employed a breast cancer murine model using eight-week-old female Balb/c mice, each weighing approximately 20 g, sourced from the School of Translational Medicine's animal laboratory at Shanghai University. To create a tumor model, 3×10^5^ TUBO cells were inoculated into the right hind limb of each mouse. The experimental design included a control group receiving saline and various treatment groups (PR, Pha-dark, Pha-light, PPC-dark, PPC-light). Drug administration commenced once tumors reached a volume of 60 mm^3^, with treatments applied bi-daily using a 660 nm laser (250 mM/cm^2^ power density, 6 min exposure, and total light dose of 90 J/cm^2^). Mice were monitored bi-daily for changes in weight and tumor volume. Following 5 treatment cycles, mice were euthanized via cervical dislocation. Organs such as the heart, liver, spleen, lungs, and kidneys were harvested for H&E staining to evaluate potential drug-induced damage. Tumor specimens underwent HER2 immunofluorescence staining, H&E staining, and Tunel immunohistochemistry to assess the therapeutic effects. To investigate T cell-mediated immune responses *in vivo*, tumor and lymphoid tissues were extracted and processed under sterile conditions with approximately 900 mg of material. Tissues were digested with Type II collagenase at 37°C for 50 min, then strained through a 75 μm cell strainer to remove aggregates. Following PBS washes, single-cell suspensions were prepared, and lymphocytes were isolated using a TBD LTS1092PK mouse splenic lymphocyte isolation kit. After additional filtering to remove debris, cells were suspended in PBS, adjusting the concentration to 1.5×10^5^ cells in 300 μL. Lymphocytes were labeled with CD80^+^/CD86^+^ primary antibodies, while tumor cells were stained with antibodies against CD8^+^/CD4^+^ and Foxp3^+^/CD4^+^. Following staining, cells were incubated at room temperature for 25 min, washed, and prepared for flow cytometry analysis using a Beckman Coulter CytoFLEX system.

**Spectral**


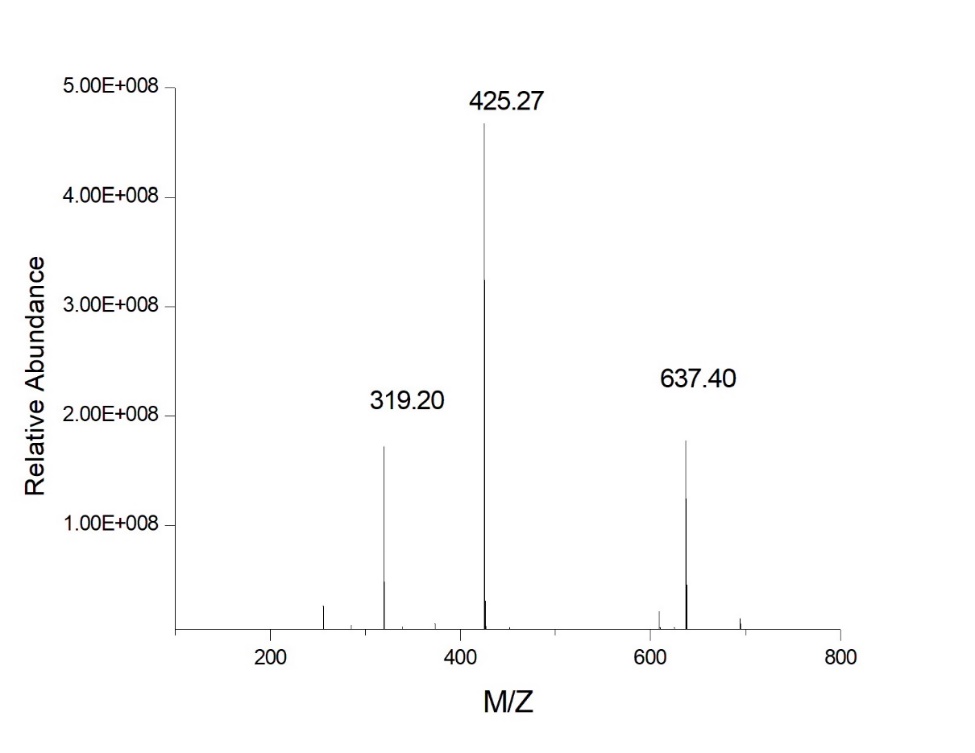


**Figure S15** States that the molecular weight of RTYGKRPKIR is 636.


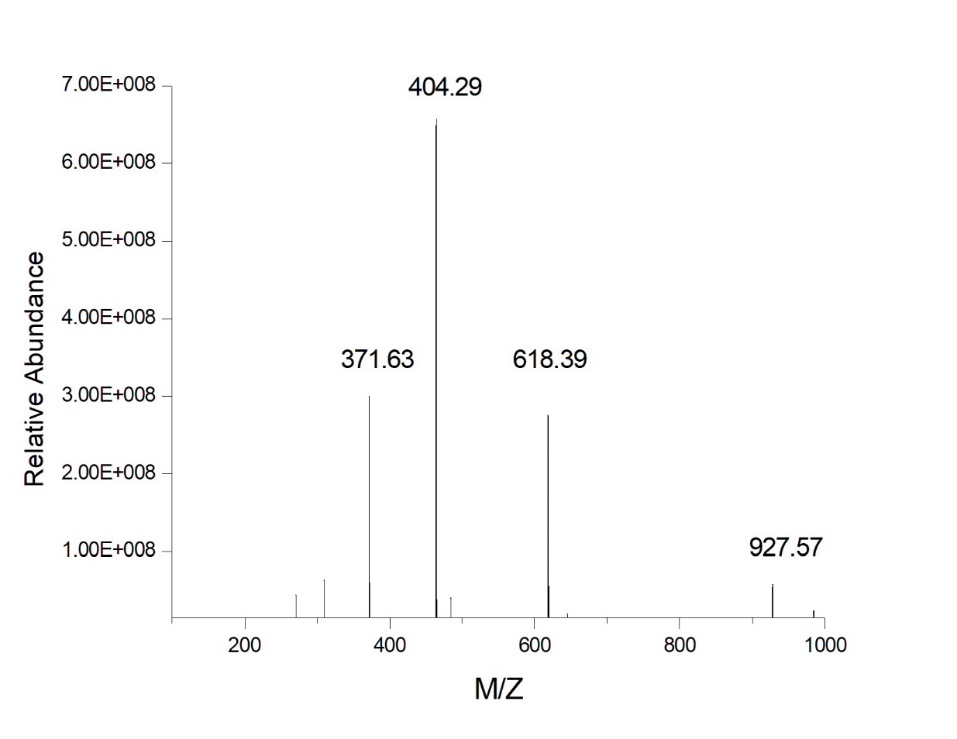


**Figure S16** Mass spectrometry analysis of the compound PR. The compound PR with the sequence PLGVRGRTYGKRPKIR has a molecular weight of 1854.


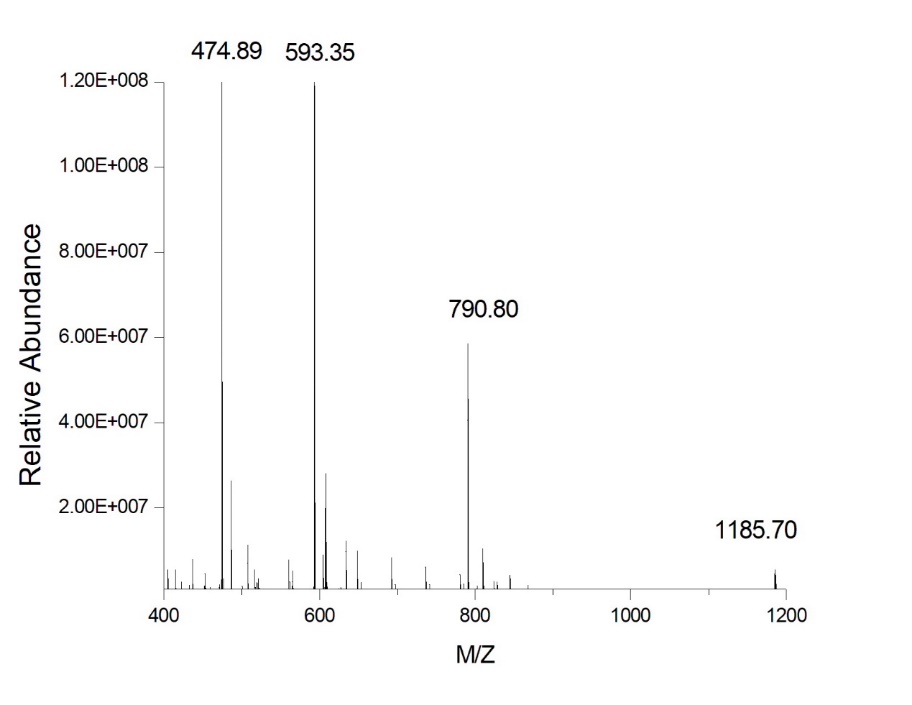


**Figure S17** Mass spectrometry results of the compound “Pha-PLGVRGRTYGKRPKIR”.The monomeric molecular weight of the compound peptide is 2370.

**REFERENCES**

[1] a) B. Merrifield, *Science* **1986**, *232*, 341-347; b) U. Boas, J. Brask, K. J. Jensen, *Chem. Rev.* **2009**, *109*, 2092-2118.

[2] D. Zhang, G. B. Qi, Y. X. Zhao, S. L. Qiao, C. Yang, H. Wang, *Adv. Mater.* **2015**, *27*, 6125-6130.
